# LncRNA XR_001779380 Primes Epithelial Cells for IFN-γ-Mediated Gene Transcription and Facilitates Age-Dependent Intestinal Antimicrobial Defense

**DOI:** 10.1128/mBio.02127-21

**Published:** 2021-09-07

**Authors:** Ai-Yu Gong, Yang Wang, Min Li, Xin-Tian Zhang, Silu Deng, Jessie M. Chen, Eugene Lu, Nicholas W. Mathy, Gislaine A. Martins, Juliane K. Strauss-Soukup, Xian-Ming Chen

**Affiliations:** a Department of Microbial Pathogens and Immunity, Rush University Medical Center, Chicago, Illinois, USA; b Department of Medical Microbiology and Immunology, Creighton University School of Medicine, Omaha, Nebraska, USA; c Deptartments of Medicine and Biomedical Sciences, Research Division of Immunology Cedars-Sinai Medical Center, David Geffen School of Medicine, University of California, Los Angeles, Los Angeles, California, USA; d Department of Chemistry, Creighton University College of Arts and Sciences, Omaha, Nebraska, USA; School of Medicine, Oregon Health & Science University

**Keywords:** *Cryptosporidium*, lncRNAs, gastrointestinal infection, interferon gamma, histone methylation, Prdm1, Swi/Snf, Pias1, innate immunity

## Abstract

Interferon (IFN) signaling is key to mucosal immunity in the gastrointestinal tract, but cellular regulatory elements that determine interferon gamma (IFN-γ)-mediated antimicrobial defense in intestinal epithelial cells are not fully understood. We report here that a long noncoding RNA (lncRNA), GenBank accession no. XR_001779380, was increased in abundance in murine intestinal epithelial cells following infection by *Cryptosporidium*, an important opportunistic pathogen in AIDS patients and a common cause of diarrhea in young children. Expression of XR_001779380 in infected intestinal epithelial cells was triggered by TLR4/NF-κB/Cdc42 signaling and epithelial-specific transcription factor Elf3. XR_001779380 primed epithelial cells for IFN-γ-mediated gene transcription through facilitating Stat1/Swi/Snf-associated chromatin remodeling. Interactions between XR_001779380 and Prdm1, which is expressed in neonatal but not adult intestinal epithelium, attenuated Stat1/Swi/Snf-associated chromatin remodeling induced by IFN-γ, contributing to suppression of IFN-γ-mediated epithelial defense in neonatal intestine. Our data demonstrate that XR_001779380 is an important regulator in IFN-γ-mediated gene transcription and age-associated intestinal epithelial antimicrobial defense.

## INTRODUCTION

Epithelial cells along the mucosal surface provide the front line of defense against luminal pathogen infection in the gastrointestinal tract. Upon microbial challenge, intestinal epithelial cells quickly initiate a series of defense responses, including production of antimicrobial molecules and release of inflammatory chemokines/cytokines. These chemokines/cytokines of epithelial cell origin may mobilize and activate immune effector cells to the infection sites. Molecular mechanisms regulating epithelial signaling pathways for this communication network are not fully understood. Large-scale transcriptome studies have revealed that transcription of protein-coding genes is far outweighed by the production of noncoding RNAs (ncRNAs), including thousands of long ncRNAs (lncRNAs) (1, 2). The expression of many lncRNAs is highly cell type specific, perhaps even more so than that of lineage-determining proteins (3, 4). This specificity appears to be tightly regulated given that expression patterns and lncRNA promoter sequences are conserved across evolutionary time (4). Physically, lncRNAs can interact with DNA, other RNAs, and proteins, either through nucleotide base pairing or via formation of structural domains generated by RNA folding (5). These properties endow lncRNAs with a versatile range of capabilities to modulate gene expression, which is only beginning to be appreciated (5). Under normal circumstances, most lncRNAs are expressed at a basal level and may be involved in maintaining cellular function (6). Many lncRNAs have been found to be targets of inflammatory pathways, and, consequently, their expression profile is altered in various cell types during inflammation or microbial infection. LncRNAs are differentially regulated in virus-infected cells (7), in host cells following parasitic infection (8), and in dendritic cells or macrophages following stimulation by ligands for Toll-like receptor 4 (TLR4) and TLR2 (9). We previously demonstrated that a long intergenic ncRNA, lincRNA-Cox2, one of the most highly induced lncRNAs in macrophages, regulates inflammatory gene transcription in intestinal epithelial cells through modulating ATP-dependent chromatin remodeling (10, 11).

*Cryptosporidium*, a protozoan parasite that infects the gastrointestinal epithelium and other mucosal surfaces in humans and animals, is an important opportunistic pathogen in patients with AIDS (12, 13). *Cryptosporidium* is also a common cause of diarrhea in young children in developing countries. After rotavirus, *Cryptosporidium* is the most common pathogen responsible for moderate-to-severe diarrhea in children younger than 1-year-old in developing countries (14). Infection with *Cryptosporidium* shows significant association with mortality in young children and appears to predispose them to lasting deficits in body growth and cognitive development (14–17). The majority of human cryptosporidial infections are caused by two species, C. parvum and C. hominis (12, 13). Humans are infected by ingesting *Cryptosporidium* oocysts. After oocyst excystation in the gastrointestinal tract to release infective sporozoites, each sporozoite then attaches to the apical membrane of intestinal epithelial cells and forms an intracellular but extracytoplasmic parasitophorous vacuole (12, 18). Therefore, epithelial cells provide the first line of defense and play a critical role in the initiation, regulation, and resolution of both innate and adaptive immune reactions (19). Indeed, the invasion of intestinal epithelial cells by C. parvum activates TLR4/nuclear factor kappa B (NF-κB) signaling, resulting in the production and secretion of various cytokines, chemokines, and antimicrobial peptides (20, 21). Interferon gamma (IFN-γ) from NK cells and other immune cells infiltered at the infection sites is key to mucosal anti-C. parvum defense (22–26). However, the key cellular regulatory elements that determine IFN-γ-mediated intestinal epithelial anti-*Cryptosporidium* defense, as well as its association with the high susceptibility of infection in AIDS patients and young children, have not been fully elucidated (12, 13).

In this study, we report that GenBank accession no. XR_001779380, a lncRNA transcript from the *2610027F03Rik* gene (27), was increased in abundance in murine intestinal epithelial cells following infection by *Cryptosporidium.* Transcription of XR_001779380 is controlled by TLR4/NF-κB signaling and by epithelial-specific transcription factor Elf3 (E74-like factor epithelial-specific transcription factor 3). XR_001779380 primes intestinal epithelial cells for IFN-γ-mediated gene transcription and antimicrobial defense, a process that is suppressed in neonatal intestinal epithelium through its interactions with PR/SET domain 1 (Prdm1). Our data support that XR_001779380 may be an important regulator in IFN-γ-mediated and age-associated intestinal epithelial antimicrobial defense.

## RESULTS

### XR_001779380 is increased in abundance in intestinal epithelium following *Cryptosporidium* infection or LPS stimulation.

**FIG 1:**
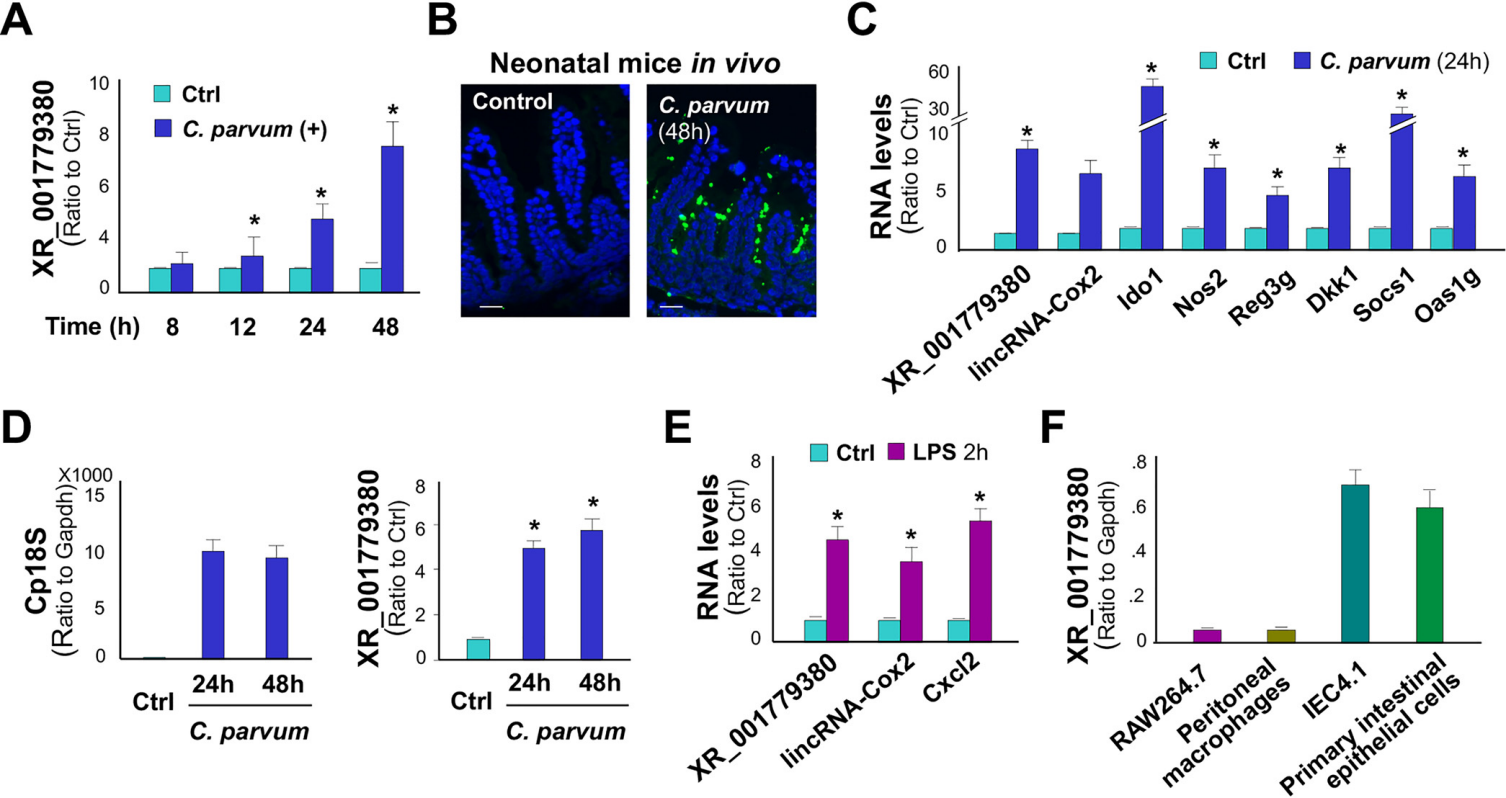
Expression of XR_001779380 and its induction in intestinal epithelial cells following C. parvum infection or LPS stimulation. (A) Expression level of XR_001779380 in IEC4.1 cells following C. parvum infection was measured using quantitative real-time PCR (qRT-PCR). (B) C. parvum infection of intestinal epithelium in neonatal mice. Indirect immunofluorescent staining of ileum confirms C. parvum infection. (C) Expression levels of XR_001779380 and lincRNA-Cox2 and several inflammatory genes in the isolated ileum epithelium following C. parvum infection *in vivo* were measured using qRT-PCR. (D) *Ex vivo* infection of enteroids by C. parvum. Infection was measured by quantification of Cp18S with qRT-PCR. Upregulation of XR_001779380 was detected in enteroids following C. parvum infection. (E) Upregulation of XR_001779380, lincRNA-Cox2, and C-X-C motif chemokine ligand (Cxcl2) in IEC4.1 cells following LPS stimulation. (F) Expression levels of XR_001779380 in murine cell lines (IEC4.1 and RAW264.7 cells) and primary cells (peritoneal macrophages and neonatal intestinal epithelial cells). Data are shown as the means ± SD from at least three independent experiments. Statistical significance (two-tailed unpaired *t* test): *, *P* < 0.05 versus noninfection or non-LPS-treated controls. Scale bars, 50 μm.

We previously performed a genome-wide transcriptome analysis of IEC4.1 cells following C. parvum infection for 24 h (8). IEC4.1 cells are transformed but nontumorigenic intestinal epithelial cells from neonatal mice (28). Infected IEC4.1 cells demonstrated a significant alteration in lncRNA expression profile (GEO accession no. GSE112247) (8). One of the top induced lncRNAs was the noncoding transcript XR_001779380 from the *2610027F03Rik* gene (27) (Fig. S1 in the supplemental material), which was increased 2.00 ± 0.12-fold in infected cells versus noninfected control (*P* < 0.001) (8). An increased level of XR_001779380 expression was further confirmed using quantitative real-time PCR (qRT-PCR) in IEC4.1 cells following C. parvum infection (Fig. 1A). Using a well-documented model of intestinal cryptosporidiosis in neonatal mice through oral administration of C. parvum oocysts (29, 30), we detected infection of C. parvum in the villus regions of ileum from infected animals by immunofluorescent staining (Fig. 1B). Consistent with results from previous studies (20, 31), enhanced expression of inflammatory and defense genes, such as nitric oxide synthase 2 (*Nos2*), indoleamine 2,3-dioxygenase 1 (*Ido1*), colony-stimulating factor 2 (*Csf2*), regenerating islet-derived 3 gamma (*Reg3g*), dickkopf WNT signaling pathway inhibitor 1 (Dkk1), suppressor of cytokine signaling 1 (Socs1), and 2′-5′ oligoadenylate synthetase 1G (Oas1g) was confirmed in the ileum epithelium of infected neonates compared with that of the noninfected control (Fig. 1C). Induction of XR_001779380 was detected in infected ileum epithelium by qRT-PCR (Fig. 1C). LincRNA-Cox2, a previously identified lncRNA induced in intestinal epithelial cells following C. parvum infection (10), was measured for control. Moreover, using an *ex vivo* infection model employing enteroids from neonatal mice (32), we detected C. parvum infection and induction of XR_001779380 in enteroids following C. parvum infection (Fig. 1D).

10.1128/mBio.02127-21.1 FIG S1 Chromosome location of the murine *2610027F03Rik* gene. The murine *2610027F03Rik* gene is localized at Chr1:122581992-122660144 (mm9) with 5 putative exons. Sequence of the XR_001779380 transcript of the expressing construct is shown. Download FIG S1, TIF file, 0.3 MB. Copyright © 2021 Gong et al. 2021 Gong et al. https://creativecommons.org/licenses/by/4.0/ This content is distributed under the terms of the Creative Commons Attribution 4.0 International license.

Increased abundance of XR_001779380 and lincRNA-Cox2 was further detected in IEC4.1 cells following lipopolysaccharide (LPS) stimulation (Fig. 1E). Although XR_001779380 is highly expressed in the central neuron system (27), it appears that it is an intestinal epithelial cell-enriched lncRNA. In our previous studies (8, 10, 11, 33), we performed several genome-wide transcriptome analyses on IEC4.1 cells and two murine macrophage cell lines (BV2 microglia and RAW264.7 cells). Comparative analysis of XR_001779380 in these cells revealed that the basal expression level of XR_001779380 was 15.54 ± 2.46-fold higher in IEC4.1 cells than that in BV2 cells and 14.17 ± 3.72-fold higher than that in RAW264.7 cells. The enriched expression of XR_001779380 in intestinal epithelial cells was further confirmed in primary peritoneal macrophages and intestinal epithelium from neonatal mice by using qRT-PCR (Fig. 1F).

### XR_001779380 promotes IFN-γ-mediated epithelial anti-*Cryptosporidium* defense.

**FIG 2:**
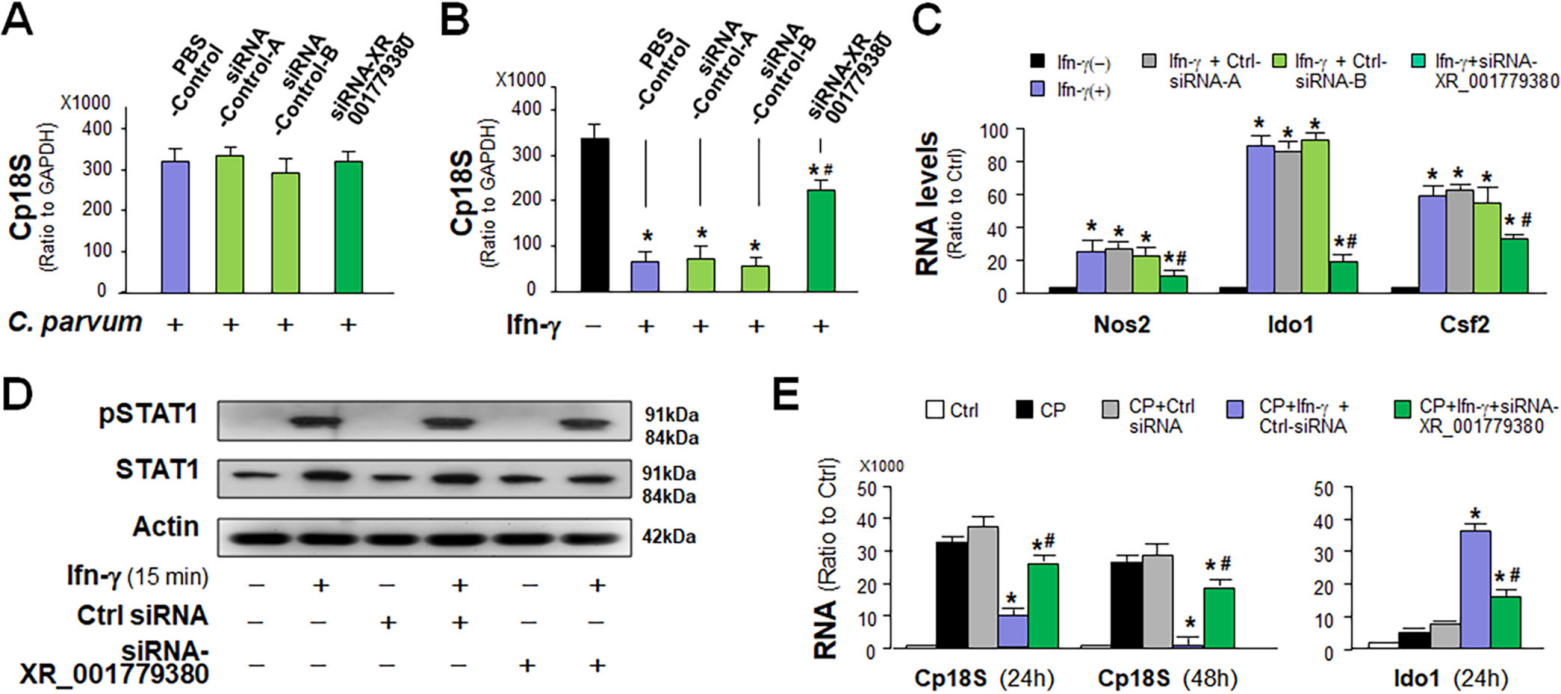
Upregulation of XR_001779380 promotes IFN-γ-mediated epithelial antimicrobial defense. (A) Impact of siRNA knockdown of XR_001779380 on C. parvum infection burden in intestinal epithelial cells. Knockdown of XR_001779380 in IEC4.1 cells showed no detectable effects on C. parvum infection burden. (B) siRNA knockdown of XR_001779380 on IFN-γ-mediated epithelial anti-C. parvum defense. Pretreatment of IEC4.1 cells with IFN-γ decreased C. parvum infection burden. Treatment of IEC4.1 cells with the siRNA to XR_001779380, but not the nonspecific siRNA controls, attenuated the inhibitory effects of IFN-γ pretreatment on C. parvum infection burden. (C) Knockdown of XR_001779380 attenuated IFN-γ-induced expression of Ido1, Nos1, and Csf2 in IEC4.1 cells as assessed using qRT-PCR. (D) Knockdown of XR_001779380 did not alter Stat1 phosphorylation in IEC4.1 cells induced by IFN-γ. Representative gel images from at least three independent experiments are shown. (E) IFN-γ treatment decreased C. parvum infection burden and induced the expression of Ido1 in enteroids, which are attenuated by siRNA knockdown of XR_001779380. Data are shown as the means ± SD from at least three independent experiments. Statistical significance (ANOVA test): *, *P* < 0.01 versus noninfection or non-IFN-γ-treated control; #, *P < *0.01 versus IFN-γ- and control siRNA-treated group.

Due to the “minimally invasive” nature of *Cryptosporidium* infection, epithelial cells play a central role in activating and orchestrating host immune responses (19). We asked whether XR_001779380 can modulate host antiparasite defense. We took the RNA interference (RNAi) approach to knockdown XR_001779380 in IEC4.1 cells and then exposed them to C. parvum infection for 24 h. Two separate nonspecific scrambled small interfering RNAs (siRNAs) were used as the control. While siRNA treatment decreased the expression of each targeted lncRNA and significantly attenuated its induction in infected cells (Fig. S2), siRNA knockdown of XR_001779380 revealed no significant effect on the parasite infection burden (Fig. 2A).

10.1128/mBio.02127-21.2 FIG S2 siRNA treatment decreased the expression of each targeted lncRNA and significantly attenuated its induction in infected cells. Knockdown of XR_001779380 expression with the siRNA in IEC4.1 cells in response to C. parvum infection. Cells were treated with the siRNA to XR_001779380 for 24 h and then exposed to C. parvum infection for 24 h. Expression level of XR_001779380 was validated by using qRT-PCR. Two nonspecific scrambled siRNAs were used for control (Ctrl-siRNA-A and Ctrl-siRNA-B). Data are shown as the means ± SD from at least three independent experiments. Statistical significance (ANOVA test): *, *P* < 0.05 versus Ctrl-siRNA-A; #, *P* < 0.05 versus Ctrl-siRNA-A + *C. parvum*. Download FIG S2, TIF file, 0.3 MB. Copyright © 2021 Gong et al. 2021 Gong et al. https://creativecommons.org/licenses/by/4.0/ This content is distributed under the terms of the Creative Commons Attribution 4.0 International license.

IFN-γ represents a key effector molecule for epithelial anti-C. parvum defense (19, 22). Consistent with previous reports (22), we detected a decreased infection burden in IEC4.1 cells pretreated with IFN-γ (Fig. 2B). Intriguingly, knockdown of XR_001779380 in IEC4.1 cells resulted in significant suppression of IFN-γ-mediated epithelial defense, reflected by a higher infection burden in XR_001779380 siRNA-treated and IFN-γ-primed cells compared than that in IFN-γ-primed cells treated with the nonspecific siRNA controls (Fig. 2B). We then measured cell response to IFN-γ stimulation using IEC4.1 cells transfected with the siRNA to XR_001779380 or nonspecific siRNA controls. Induced expression of selected IFN-γ-controlled defense genes, *Nos2*, *Ido1*, and *Csf2*, was attenuated in cells transfected with the siRNA to XR_001779380 (Fig. 2C). This inhibitory effect is specific to XR_001779380 siRNA knockdown, as two nonspecific siRNA controls had no impact on IFN-γ-mediated gene transcription (Fig. 2C). Suppression of these genes by XR_001779380 siRNA is at the transcriptional level, as their mRNA stability was not altered by XR_001779380 siRNA knockdown (Fig. S3). In addition, the XR_001779380 siRNA had no significant effect on the cytoplasmic activation of the Stat1 pathway triggered by IFN-γ, as there was no significant difference in the cytoplasmic Stat1 phosphorylation in siRNA-treated IEC4.1 cells (Fig. 2D). Complementarily, overexpression of XR_001779380 resulted in enhanced IFN-γ-mediated inhibition of infection burden in IEC4.1 cells (Fig. S4). Accordingly, a further increase of IFN-γ-controlled defense gene transcription was detected in XR_001779380-overexpressed cells in response to IFN-γ stimulation (Fig. S4). Moreover, a higher infection burden was detected in XR_001779380 siRNA-treated and IFN-γ-primed enteroids than that in IFN-γ-primed enteroids treated with the nonspecific siRNA controls (Fig. 2E). The expression level of Ido1 induced by IFN-γ was significantly reduced in the XR_001779380 siRNA-treated and C. parvum-infected enteroids compared with that in infected enteroids treated with the nonspecific siRNA control (Fig. 2E). These data suggest that XR_001779380 promotes IFN-γ-mediated gene transcription and epithelial cell anti-*Cryptosporidium* defense.

10.1128/mBio.02127-21.3 FIG S3 Impact of XR_001779380 siRNA knockdown on Nos2, Ido1, and Csf2 RNA stability. IEC4.1 cells were transfected with the siRNA to XR_001779380 for 24 h and then exposed to IFN-γ for 4 h, followed by treatment of actinomycin D to block transcription. At 0, 2, 4, and 6 h after actinomycin D treatment, cells were collected, and expression levels of Nos2, Ido1, and Csf2 were measured. Cells transfected with the nonspecific siRNA were used as the control. Data represent three independent experiments. Download FIG S3, TIF file, 0.3 MB. Copyright © 2021 Gong et al. 2021 Gong et al. https://creativecommons.org/licenses/by/4.0/ This content is distributed under the terms of the Creative Commons Attribution 4.0 International license.

10.1128/mBio.02127-21.4 FIG S4 Induction of XR_001779380 on intestinal epithelial anti-C. parvum defense and IFN-γ-mediated gene transcription in intestinal epithelial cells. (A) Overexpression of XR_001779380 in intestinal epithelial cells. IEC4.1 cells were treated with the vector expressing XR_001779380 for 24 h and then exposed to C. parvum infection for additional 8 h. Expression levels of XR_001779380 were measured. Cells transfected with the empty vector were used as control. *, *P* < 0.01 versus noninfection; #, *P* < 0.01 versus empty vector-transfected cells. (B) Overexpression of XR_001779380 in intestinal epithelial cells resulted in a significantly lower infection burden of C. parvum. IEC4.1 cells were treated with the vector expressing XR_001779380 for 24 h and then exposed to C. parvum infection for additional 8 h in the presence of IFN-γ. Infection burden was measured in cells following infection for 24h. *, *P* < 0.05 versus non-IFN-γ-treated control; #, *P* < 0.05 versus IFN-γ-treated cells transfected with the empty vector. (C) Overexpression of XR_001779380 promoted IFN-γ-mediated gene transcription in intestinal epithelial cells. IEC4.1 cells IEC4.1 cells were treated with the vector expressing XR_001779380 for 24 h and then exposed to IFN-γ stimulation for additional 4 h. Expression levels of selected genes were measured, and data represent three independent experiments. Statistical significance (ANOVA test): * *P* < 0.05 versus non-IFN-γ-treated control; #, *P* < 0.05 versus IFN-γ-treated cells transfected with the empty vector. Download FIG S4, TIF file, 0.4 MB. Copyright © 2021 Gong et al. 2021 Gong et al. https://creativecommons.org/licenses/by/4.0/ This content is distributed under the terms of the Creative Commons Attribution 4.0 International license.

### Expression of XR_001779380 is controlled by TLR4/NF-κB/Cdc42 signaling and epithelial-specific transcription factor Elf3 in infected intestinal epithelial cells.

**FIG 3:**
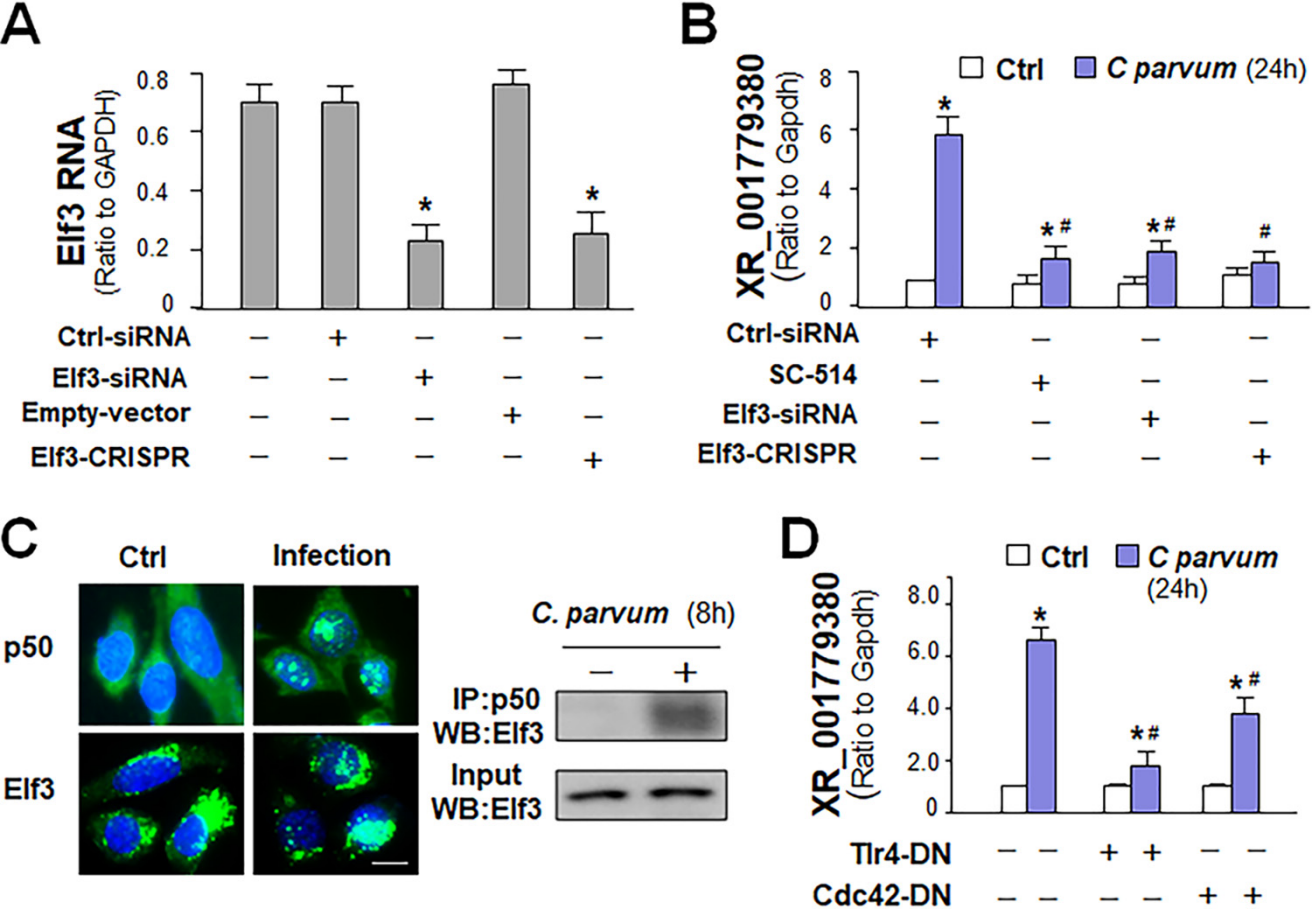
Transcription of XR_001779380 from the *2610027F03Rik* gene through activation of TLR4/NF-кB/Cdc42/Elf3 signaling. (A) Knockdown of Elf3 using the RNAi interference and CRISPR/Cas9 approaches in IEC4.1 cells. (B) Knockdown of Elf3, as well as inhibition of NF-кB signaling by SC-514, inhibited the induction of XR_001779380 in IEC4.1 cells induced by C. parvum infection. (C) Nuclear translocation of NF-кB p50 and Elf3 in IEC4.1 cells following infection by fluorescence microscopy and physical association between p50 and Elf3 in infected cells as assessed using coimmunoprecipitation. Representative gel images from at least three independent experiments are shown. (D) Knockdown of either TLR4 or Cdc42 inhibited the induction of XR_001779380 in IEC4.1 cells by C. parvum infection. Data are shown as the means ± SD from at least three independent experiments. Statistical significance (ANOVA test): *, *P* < 0.01 versus noninfection control. Scale bars, 5 μm.

The invasion of intestinal epithelial cells by C. parvum *in vitro* activates the TLR4/NF-κB signal pathway to trigger host defense responses (34). Because XR_001779380 can be induced by C. parvum infection and LPS stimulation, we asked whether TLR4/NF-κB signaling is required for XR_001779380 induction. In addition, it is possible that epithelial-specific transcription factors, such as Elfs, caudal type homeobox 1 (CDX1), and CDX2 (35, 36), may also contribute to its higher expression level in intestinal epithelial cells. Based on TFSEARCH (http://www.cbrc.jp/research/db/TFSEARCH.html) and MOTIF (http://motif.genome.jp/) database search (37, 38), we failed to detect the classical NF-κB consensus sequence in the potential regulatory regions of these epithelial lncRNA genes. Nevertheless, we detected a potential binding sequence in the promoter region of *2610027F03Rik* gene locus for Elf3 (also known as Ese-1 or Esx) (35). We then knocked down Elf3 in IEC4.1 cells using a specific siRNA or a CRISPR/Cas9 construct (Fig. 3A). Knockdown of Elf3 with either the siRNA or the CRISPR/Cas9 construct attenuated the induction of XR_001779380 by C. parvum infection (Fig. 3B). Inhibition of NF-κB signaling with an IKK2 inhibitor, SC-514 (39), also attenuated C. parvum-induced XR_001779380 expression (Fig. 3B). By using immunofluorescent microscopy, we detected nuclear translocation of both Elf3 and NF-κB p50 subunit in IEC4.1 cells following C. parvum infection (Fig. 3C). Using coimmunoprecipitation (co-IP), we observed direct interactions between Elf3 and the NF-κB p50 subunit in infected IEC4.1 cells (Fig. 3C). In our previous studies, we detected TLR4-dependent activation of NF-κB signaling (34) and activation of the cell division control protein 42 homolog (Cdc42) signaling in cells following C. parvum infection (40). Intriguingly, transfection of cells with the TLR4-dominant negative (DN) mutant or Cdc42-DN mutant attenuated C. parvum-induced XR_001779380 expression in IEC4.1 cells (Fig. 3D). Therefore, TLR4/NF-κB/Cdc42 signaling and epithelial-specific transcription factor Elf3 may coordinate XR_001779380 induction in intestinal epithelial cells in response to C. parvum infection.

### XR_001779380 facilitates Stat1-associated chromatin remodeling to promote IFN-γ-mediated transcription of defense genes in intestinal epithelial cells.

**FIG 4:**
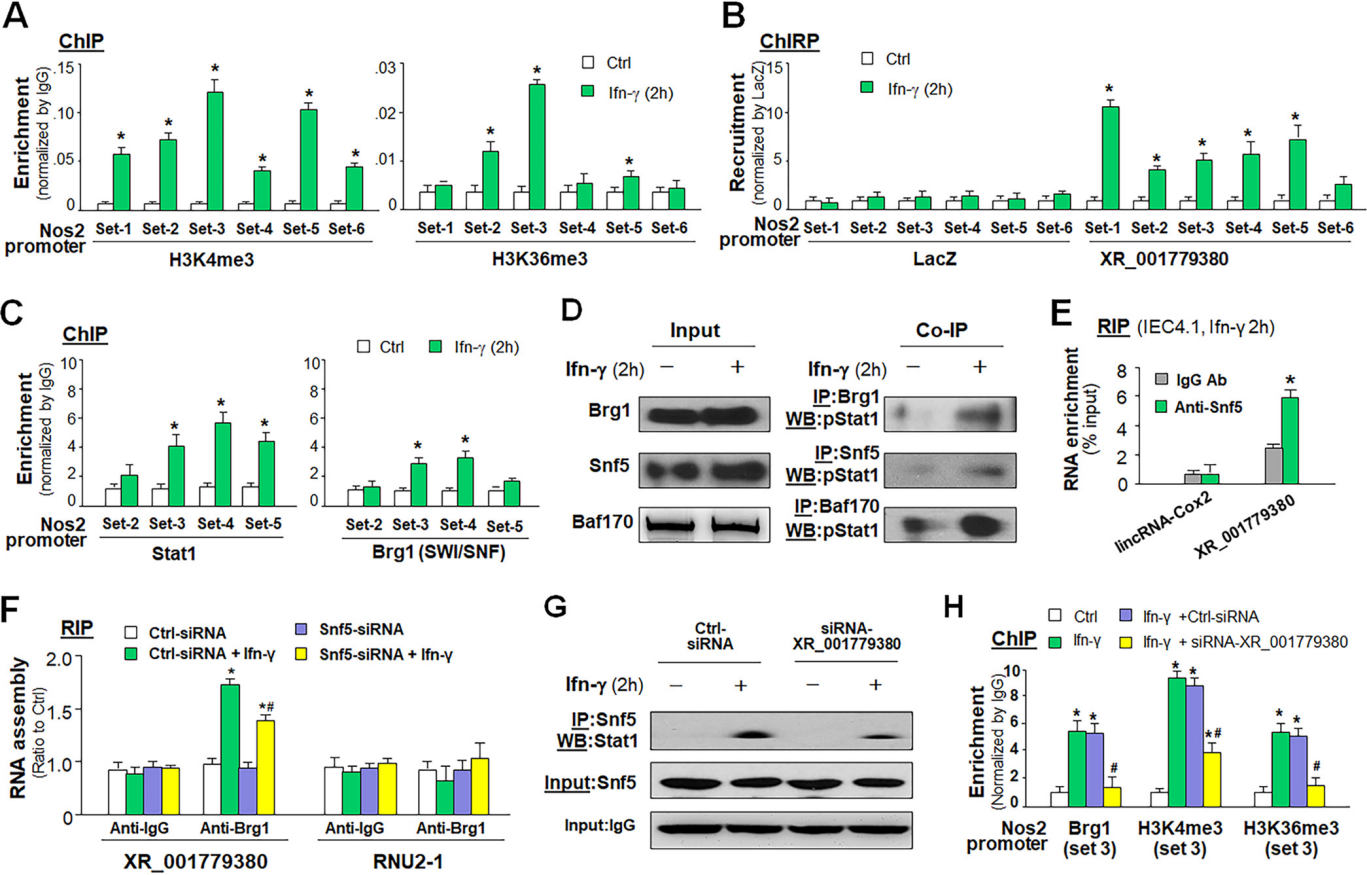
XR_001779380 modulates Stat1/Swi/Snf-associated chromatin remodeling to promote IFN-γ-mediated transcription of defense genes in intestinal epithelial cells. (A) Enrichment of H3K4me3 and H3K36me3 at the *Nos2* promoter in IEC4.1 cells following IFN-γ stimulation as assessed using ChIP analysis. H3K4me3 and H3K36me3 signal distributions at the *Nos2* promoter region were quantified by qRT-PCR. (B) Recruitment of XR_001779380 to the *Nos2* promoter in IEC4.1 cells following IFN-γ stimulation. Recruitment of XR_001779380 was assessed using ChIRP analysis with a pool of probes specific to XR_001779380 and quantified by qRT-PCR using the same primer sets as for ChIP analysis. Probes to LacZ were used as control. (C) Increased recruitment of Stat1 and Brg1 to the *Nos2* promoter in IEC4.1 cells following IFN-γ stimulation as assessed by ChIP analysis. (D) Increased physical association between pStat1 and Swi/Snf complex was detected by co-IP in IEC4.1 cells following IFN-γ stimulation. Antibodies to Baf170, Snf5 and Brg1 (components of Swi/Snf complex) were used separately for immunoprecipitation, followed by Western blotting of pStat1. (E) Assembly of XR_001779380, but not lincRNA-Cox2, to the Swi/Snf complex in IEC4.1 cells induced by IFN-γ. Anti-Snf5 Ab was used for immunoprecipitation and anti-IgG as the control for RIP analysis. (F) Knockdown of Snf5 blocked IFN-γ-induced recruitment of XR_001779380 to the Swi/Snf complex. Cells were treated with the control siRNA or Snf5-siRNA for 24 h and then stimulated with IFN-γ for 2 h, followed by RIP analysis using anti-Brg1 (anti-IgG as the control). The unrelated ncRNA RNU2-1 was measured for control. (G) siRNA knockdown of XR_001779380 blocked IFN-γ-induced assembly of Stat1 to the Swi/Snf complex in IEC4.1 cells. Cells were treated with the control siRNA or XR_001779380-siRNA for 14 h and then stimulated with IFN-γ for 2 h, followed by co-IP analysis using anti-Snf5 for immunoprecipitation and anti-Stat1 for Western blotting. Inputs were blotted with anti-Snf5 and the heavy chain of IgG in the immunoprecipitation blotting (marked as Input:IgG) was also shown for control. (H) Knockdown of XR_001779380 inhibited IFN-γ-induced recruitment of Brg1 and enrichment of H3K4me3 and HeK36me3 at the *Nos2* promoter region in IEC4.1 cells. Data are shown as the means ± SD from at least three independent experiments. Representative gel images from three independent experiments are shown in panels D and G. Statistical significance (ANOVA test): *, *P* < 0.01 versus non-IFN-γ-treated control; #, *P < *0.01 versus IFN-γ- and control siRNA-treated group.

To address the molecular mechanisms underlying XR_001779380-promoted gene transcription in intestinal epithelial cells in response to IFN-γ, we measured the enrichment of transcriptionally active histone methylations in the promoter regions of *Nos2* and *Ido1* gene loci in IEC4.1 cells following IFN-γ stimulation. Using chromatin immunoprecipitation (ChIP) analysis, we detected in IFN-γ-treated cells a significant increase of H3K36me3 and H3K4me3 at multiple sites within the regulatory promoter regions of *Nos2* (Fig. 4A) and *Ido1* (Fig. S5). Using the chromatin isolation by RNA purification (ChIRP) analysis, we detected the recruitment of XR_001779380 to several sites within the regulatory promoter regions of *Nos2* (Fig. 4B) and *Ido1* (Fig. S5) gene loci in IEC4.1 cells following IFN-γ stimulation.

10.1128/mBio.02127-21.5 FIG S5 Enrichment od H3K4me3 and H3K36me3 and recruitment of XR_001779380 to the promoter region of the *Ido1* gene in intestinal epithelial cells in response to IFN-γ stimulation. (A) Enrichment of H3K4me3 and H3K36me3 to the Ido1 promoter in IEC4.1 cells following IFN-γ stimulation for 2 h as assessed by ChIP analysis using antibodies to H3K4me3 and H3K36me3, respectively. (B) Recruitment of XR_001779380 to the Ido1 promoter in IEC4.1 cells following IFN-γ stimulation for 2 h, as assessed by ChIRP analysis using a pool of probes specific to XR_001779380. Recruitment of XR_001779380 to the Ido1 promoter region was quantified by real-time PCR using the same primer sets as for ChIP analysis. Data represent three independent experiments. Statistical significance (two-tailed unpaired *t* test): *, *P* < 0.01 versus non-IFN-γ-treated control. Download FIG S5, TIF file, 0.3 MB. Copyright © 2021 Gong et al. 2021 Gong et al. https://creativecommons.org/licenses/by/4.0/ This content is distributed under the terms of the Creative Commons Attribution 4.0 International license.

In response to IFN-γ stimulation, Stat1 forms homodimers or heterodimers with Stat3 that bind to the IFN-γ-activated sequence promoter element to trigger gene transcription (41). Increasing evidence supports that the ATP-dependent SWItch/Sucrose NonFermentable (Swi/Snf) remodeling complex may be a coregulator for IFN-γ-mediated gene transcription (42–47). Important components of the Swi/Snf complex include Brg1 (Swi/Snf-related, matrix-associated, actin-dependent regulator of chromatin, subfamily a, member 4), Snf5 (subfamily b, member 1), and Baf170 (subfamily c, member 2) (42). Recruitment of Stat1 was detected in the promoter region of the *Nos2* gene in IFN-γ-treated IEC4.1 cells (Fig. 4C). Recruitment of Brg1 was detected to the same sequence region of the *Nos2* gene in IEC4.1 cells following IFN-γ stimulation (Fig. 4C). Consistent with data from previous reports in lymphocytes and HeLa cells (44, 45), we demonstrated that Stat1 is recruited to the Swi/Snf remodeling complex in IFN-γ-treated IEC4.1 cells using specific antibodies to Brg1, Snf5, and Baf170 for co-IP analysis (Fig. 4D), suggesting that the physical association between Stat1 and the Swi/Snf complex and the subsequent chromatin remodeling may be involved in IFN-γ-mediated gene transcription in intestinal epithelial cells.

To define whether XR_001779380 is involved in this process, we tested if XR_001779380 is recruited to the Stat1/Swi/Snf complex. Of these various components of the Swi/Snf complex, Snf5 is an RNA-binding protein and thus can bind to specific lncRNAs (48) and can be assembled into the SWI/SNF complex in cells following IFN-γ stimulation (46, 47). Using anti-Snf5 for RNA immunoprecipitation (RIP) analysis, we detected the presence of XR_001779380, but not lincRNA-Cox2, in the Swi/Snf complex in IFN-γ-treated IEC4.1 cells (Fig. 4E). Knockdown Snf5 in IEC4.1 cells caused a significant decrease in the assembly of XR_001779380 to the Swi/Snf complex induced by IFN-γ (Fig. 4F). Furthermore, siRNA knockdown XR_001779380 reduced IFN-γ-induced physical association between Stat1 and the Swi/Snf complex (Fig. 4G), as well as IFN-γ-induced recruitment of Brg1 and enrichment of the active histone modifications (H3K36me3 and H3K4me3) at the promoter regions of *Nos2* (Fig. 4H) and *Ido1* (Fig. S6) gene loci. These data suggest that that XR_001779380 may act as a coregulator for Stat1-Swi/Snf interactions or their chromatin occupancy in intestinal epithelial cells in response to IFN-γ stimulation.

10.1128/mBio.02127-21.6 FIG S6 LncRNA XR_001779380 promotes Stat1/Swi/Snf-associated chromatin remodeling to promote IFN-γ-mediated transcription of the *Ido1* gene in intestinal epithelial cells. (A) Increase in the recruitment of Brg1 and Stat1 to the Ido1 promoter in IEC4.1 cells following IFN-γ stimulation for 2 h as assessed by ChIP analysis using antibodies to Stat1 or Brg1, respectively. (B) Knockdown of XR_001779380 inhibited IFN-γ-induced recruitment of Brg1 and enrichment of H3K4me3 and HeK36me3 at the Ido1 promoter region in IEC4.1 cells. IEC4.1 cells were transfected with siRNA to XR_001779380 for 24 h and then exposed to IFN-γ stimulation for 2 h. Recruitment of Brg1 and enrichment of H3K4me3 and H3K36me3 signal distributions at the Ido1 promoter region were quantified by ChIP analysis. Knockdown of XR_001779380 inhibited IFN-γ-induced recruitment of Brg1 and enrichment of H3K4me3 and HeK36me3 at the Ido1 promoter region in IEC4.1 cells. Data represent three independent experiments. Statistical significance (ANOVA test): *, *P* < 0.01 versus non-IFN-γ-treated control; #, *P* < 0.01 versus IFN-γ-treated cells transfected with the nonspecific control siRNA. Download FIG S6, TIF file, 0.3 MB. Copyright © 2021 Gong et al. 2021 Gong et al. https://creativecommons.org/licenses/by/4.0/ This content is distributed under the terms of the Creative Commons Attribution 4.0 International license.

### IFN-γ-mediated anti-*Cryptosporidium* defense is suppressed in neonatal intestinal epithelium.

**FIG 5:**
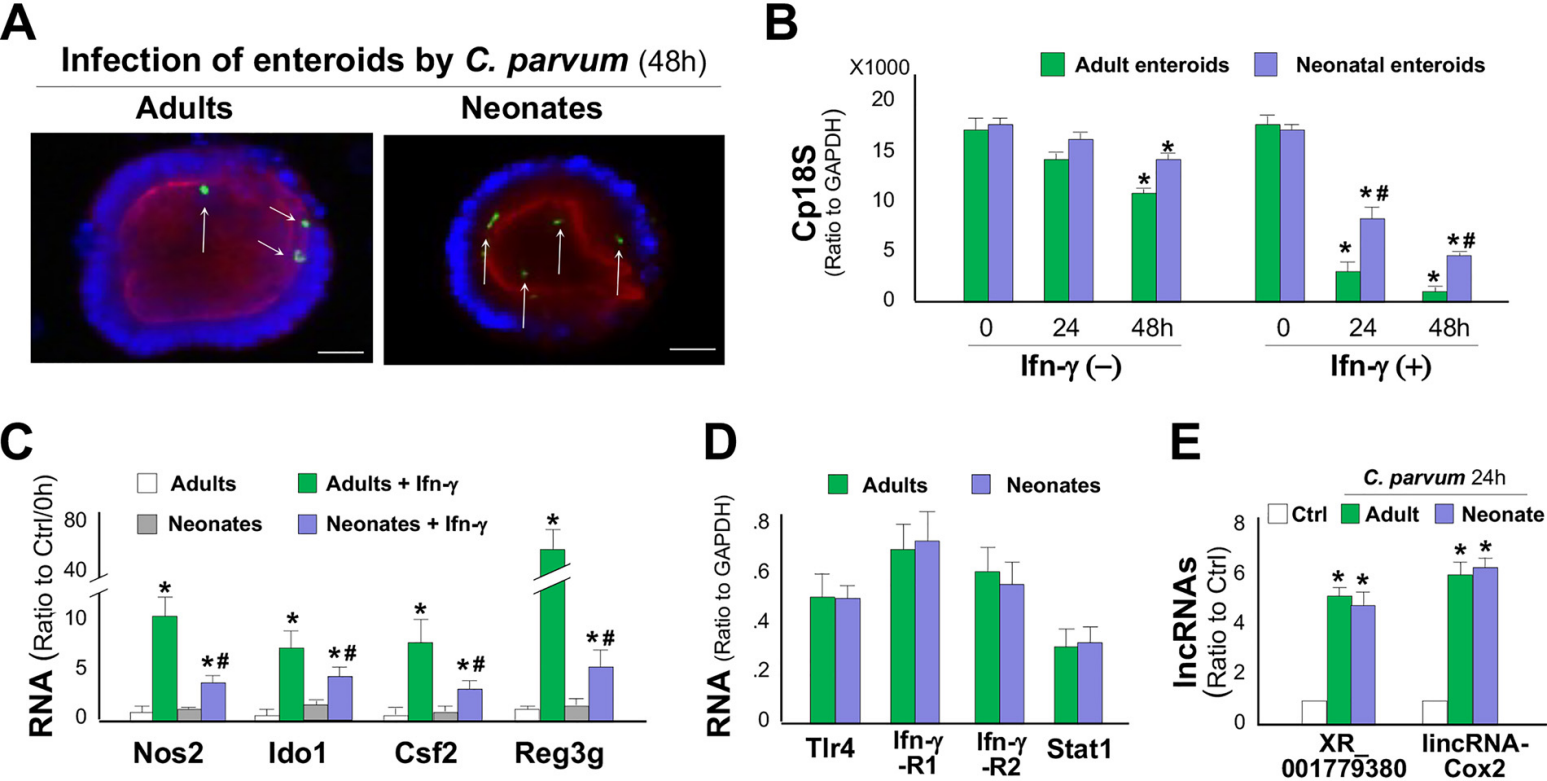
IFN-γ-mediated antimicrobial defense to C. parvum infection is suppressed in neonatal intestinal epithelium. (A) C. parvum infection in enteroids from neonatal and adult mice in the absence of IFN-γ as assessed by fluorescence microscopy (C. parvum in green by arrows). (B) C. parvum infection burden in enteroids from neonatal and adult mice. Enteroids were first infected for 8 h and then cultured for additional 24 and 48 h in the presence or absence of IFN-γ. Statistical significance (ANOVA test): *, *P* < 0.01 versus control (0 h); #, *P < *0.01 versus adult enteroids treated with IFN-γ for the same time period. (C) Expression levels of defense genes in enteroids from neonatal and adult mice following infection in the presence or absence of IFN-γ. Statistical significance (ANOVA test): *, *P* < 0.01 versus non-IFN-γ-treated control; #, *P < *0.01 versus IFN-γ-treated adult enteroids. (D) RNA levels of key signaling components in enteroids from neonatal and adult mice as assessed by qRT-PCR. (E) Upregulation of XR_001779380 and lincRNA-Cox2 in enteroids from neonatal and adult mice following C. parvum infection. Data are shown as the means ± SD from at least three independent experiments. Statistical significance (ANOVA test): *, *P* < 0.01 versus noninfection control. Scale bars, 20 μm.

While immunocompetent adult mice are resistant to *Cryptosporidium* infection, only neonatal mice and adults in immunocompromised strains are susceptible (18, 49). Deficiency in epithelial cell defense and adaptive immunity in neonates may account for the high susceptibility to C. parvum infection in neonates (18). Taking advantage of the minimal involvement of adaptive immunity in the *ex vivo* enteroid infection model, we investigated the epithelial innate defense to C. parvum infection in neonates versus in adults. When the same number of C. parvum sporozoites was applied to infect enteroids, we detected a slightly higher infection burden at 24 and 48 h after infection in enteroids from neonates than that in enteroids from adults (Fig. 5A and B). In the presence of IFN-γ, a significant decrease in the infection burden in enteroids from neonates was observed (Fig. 5B). However, a much stronger decrease in the infection burden was detected in adult enteroids after treatment with the same dose of IFN-γ (Fig. 5B). When enteroids were exposed to IFN-γ only (without C. parvum infection), a stronger response (reflected by the induction of several IFN-γ-responsive genes) was observed in enteroids from adults than that in enteroids from neonates (Fig. 5C). Interestingly, the expression levels of TLR4, IFN-γR1, IFN-γR2, and Stat1 were similar in enteroids from neonates and adults (Fig. 5D). Upregulation of epithelial cell-enriched lncRNAs, such as XR_001779380 and lincRNA-Cox2, was also similar in enteroids from neonatal and adult mice following C. parvum infection (Fig. 5E).

### Prdm1 mediates suppression of IFN-γ-mediated anti-*Cryptosporidium* defense in neonatal intestinal epithelium.

**FIG 6:**
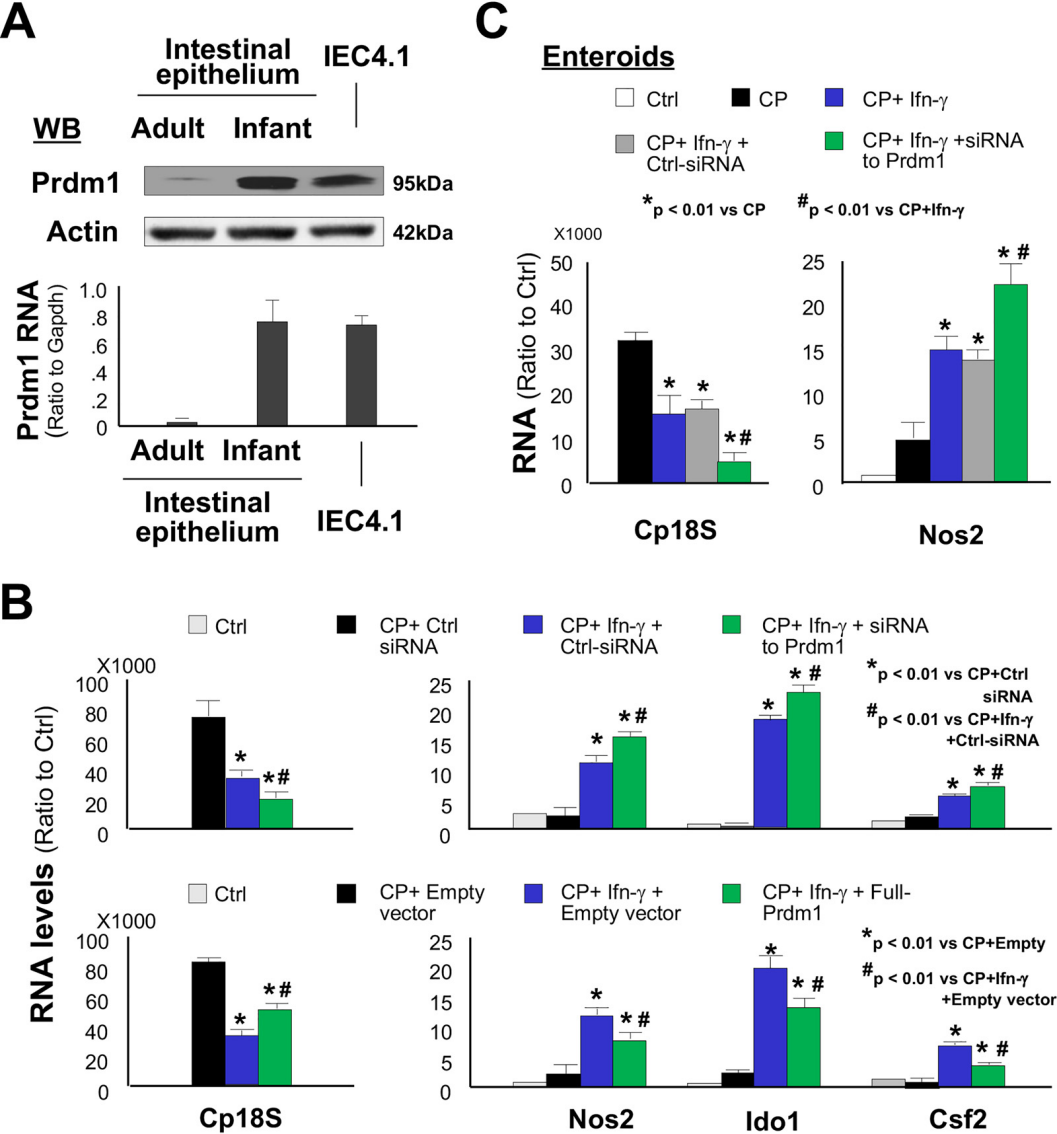
Prdm1 mediates suppression of IFN-γ-mediated antimicrobial defense in neonatal intestinal epithelium. (A) Expression of Prdm1 in primarily intestinal epithelial cells isolated from neonates, but not from adult mice, and in cultured IEC4.1 cells as measured using qRT-PCR and Western blotting. Representative gel images from at least three independent experiments are shown. (B) Effects of Prdm1 knockdown or forced Prdm1 expression on IFN-γ-mediated decrease of infection burden and IFN-γ-triggered gene expression in IEC4.1 cells in response to C. parvum infection. Statistical significance (ANOVA test): *, *P* < 0.01 versus control siRNA-treated/empty vector-transfected and C. parvum-infected cells; #, *P < *0.01 versus control siRNA-treated/empty vector-transfected and C. parvum-infected and IFN-γ-treated cells. (C) Knockdown of Prdm1 in neonatal enteroids enhanced IFN-γ-mediated antimicrobial defense, with a further decrease of infection burden (Cp18s level) and increase of Nos2 expression in IFN-γ-treated infected neonatal enteroids. Data are shown as the means ± SD from at least three independent experiments. Statistical significance (ANOVA test): *, *P* < 0.01 versus C. parvum-infected enteroids; #, *P < *0.01 versus IFN-γ-treated and C. parvum-infected enteroids.

PRDM1 (also known as Blimp1) is a transcription repressor important to cell differentiation and acts as a master regulator of intestinal epithelium maturation (50). It is strongly expressed throughout the epithelium of the embryonic gut and orchestrates orderly and extensive reprogramming of the postnatal intestinal epithelium in mice and humans (50, 51). Previous studies have identified the immunological functions of PRDM1 in various immune cell types such as T and B lymphocytes (52), including its action as a critical negative regulator of NK function (53). We then investigated a potential role for Prdm1 in regulating IFN-γ-mediated anti-C. parvum defense in intestinal epithelial cells in neonates. We consistently detected a high expression level of Prdm1, both at the mRNA and protein levels, in intestinal epithelial cells primarily isolated from neonatal mice and in cultured IEC4.1 cells (Fig. 6A). Consistent with results from previous studies (51), Prdm1 was absent in intestinal epithelial cells isolated from adult mice (Fig. 6A). Knockdown of Prdm1 in IEC4.1 cells resulted in enhanced antimicrobial activity against C. parvum infection induced by IFN-γ, with a significant further decrease in the infection burden and a further increase in the expression of defense genes, including *Nos2*, *Ido1*, and *Csf2* (Fig. 6B). Complementarily, IEC4.1 cells with forced expression of Prdm1 resulted in an increase of the infection burden and a decrease in the expression of those defense genes in infected cells in response to IFN-γ stimulation (Fig. 6B). Furthermore, knockdown of Prdm1 in cultured enteroids from neonatal mice promoted IFN-γ-mediated defense response to C. parvum infection, reflected by a further decrease in infected burden induced by IFN-γ (Fig. 6C). Accordingly, the expression level of IFN-γ-controlled *Nos2* gene was markedly enhanced in infected neonatal enteroids with the Prdm1 siRNA treatment (Fig. 6C). Due to absence of Prdm1 in adult intestine, as predicted, siRNA knockdown of Prdm1 showed no effects on IFN-γ-induced Nos2 expression in cultured enteroids from adult mice (Fig. S7). Thus, Prdm1 may suppress IFN-γ-mediated anti-*Cryptosporidium* defense in intestinal epithelial cells in neonates.

10.1128/mBio.02127-21.7 FIG S7 siRNA knockdown of Prdm1 showed no effect on IFN-γ-induced Nos2 expression in cultured enteroids from adult mice. Enteroids from adult mice were treated with the siRNA to XR_001779380 for 24 h and then treated with IFN-γ for additional 2 h. Expression level of Nos2 was validated by using qRT-PCR. A nonspecific scrambled siRNA was used as the control (Ctrl-siRNA). Data are shown as the means ± SD from at least three independent experiments. Statistical significance (ANOVA test): *, *P* < 0.01 versus control. Download FIG S7, TIF file, 0.2 MB. Copyright © 2021 Gong et al. 2021 Gong et al. https://creativecommons.org/licenses/by/4.0/ This content is distributed under the terms of the Creative Commons Attribution 4.0 International license.

### Prdm1 interacts with XR_001779380 to attenuate Swi/Snf-mediated anti-*Cryptosporidium* defense induced by IFN-γ in neonatal intestinal epithelium.

**FIG 7:**
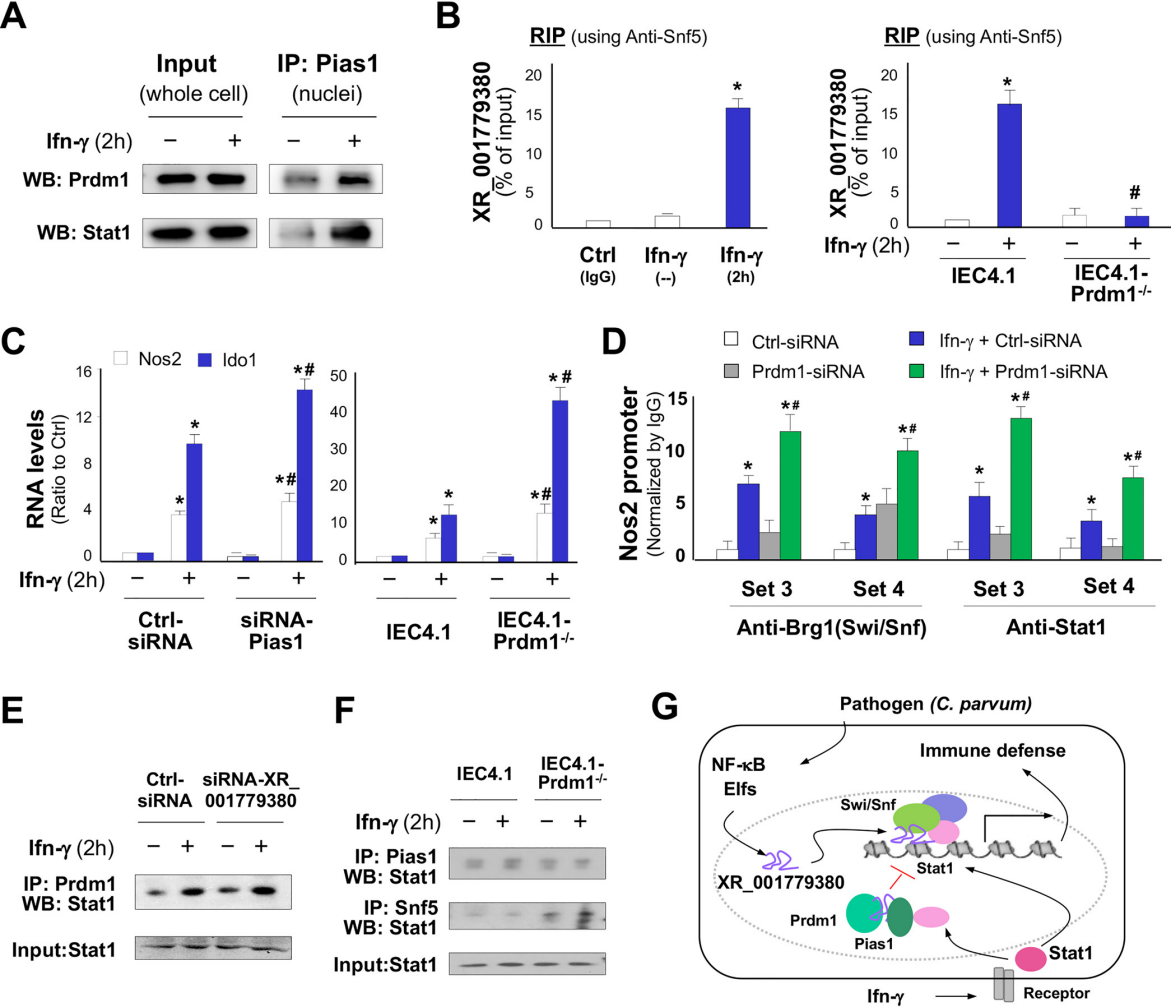
Prdm1 interacts with XR_001779380 to attenuate IFN-γ-mediated antimicrobial defense in neonatal intestinal epithelium. (A) Physical association of Prdm1 with Pias1 in the nuclei extracts of IEC4.1 cells induced by IFN-γ, as measured by co-IP. Whole-cell extracts were used as the input control. (B) Assembly of XR_001779380 to the Prdm1/Pias1 complex induced by IFN-γ in IEC4.1 cells, as assessed by RIP assay with anti-Pias1 (anti-IgG as the control). IFN-γ-induced assembly of XR_001779380 to the Prdm1/Pias1 complex was not detected in IEC4.1-Prdm1^−/−^ cells. (C) Knockdown of Pias1 or Prdm1 in IEC4.1 cells resulted in an increased expression of Nos2 and Ido1 in response to IFN-γ stimulation. (D) siRNA knockdown of Prdm1 resulted in a significant increase in the recruitment of the Swi/Snf/Stat1 complex to the *Nos2* promoter region in IEC4.1 cells in response to IFN-γ, as revealed by ChIP analysis using anti-Brg1 or anti-Stat1. (E) Knockdown of XR_001779380 showed no effects on the assembly of Stat1 to the Prdm1/Pias1 complex, as revealed by co-IP analysis using anti-Prdm1 for immunoprecipitation and anti-Stat1 for Western blotting. (F) Knockdown of Prdm1 in IEC4.1 cells decreased the assembly of Stat1 to the Pias1 complex but increased the assembly of Stat1 to the Swi/Snf complex in response to IFN-γ stimulation. IEC4.1 and IEC4.1-Prdm1^−/−^ cells were treated IFN-γ for 2 h, followed by co-IP analysis using anti-Pias1 or anti-Snf5 for immunoprecipitation and anti-Stat1 for Western blotting, respectively. (G) Model of XR_001779380 in IFN-γ-induced epithelial cell antimicrobial defense. Induction of XR_001779380 primes epithelial cells for IFN-γ-mediated antimicrobial gene transcription through modification of Stat1/Swi/Snf-mediated chromatin remodeling. Prdm1 may suppress IFN-γ-mediated antimicrobial defense through its interaction with Pias1 and XR_001779380, contributing to the higher susceptibility to infection in infants. Data are shown as the means ± SD from at least three independent experiments. Representative gel images from three independent experiments are shown in panels A, E, and F. Statistical significance (ANOVA test): *, *P* < 0.01 versus non-IFN-γ-treated control; #, *P < *0.01 versus IFN-γ-treated group.

Prdm1 can interact with protein inhibitor of activated Stat1 (Pias1, also called as Ddxbp1) to modulate Stat1-mediated gene transcription (54). In addition, Prdm1 is an RNA-binding protein, and its PR zinc finger domain mediates specific interactions with RNA molecules (50, 55). We detected Prdm1 in the Pias1/Stat1 complex immunoprecipitated from IEC4.1 cells following IFN-γ stimulation (Fig. 7A). We also detected the assembly of XR_001779380 to the Prdm1/Pias1 complex in IEC4.1 cells following IFN-γ stimulation using RIP analysis (Fig. 7B). Such IFN-γ-induced assembly of XR_001779380 to the Prdm1/Pias1 complex was not detected in IEC4.1 cells deficient with Prdm1 (Fig. 7B). Knockdown of Pias1 with a specific siRNA or knockout of Prdm1 using the CRISPR/Cas9 approach in IEC4.1 cells resulted in a significant increase of IFN-γ-induced expression level of Nos2 and Ido1 (Fig. 7C). An increase in the recruitment of the Swi/Snf complex and Stat1 to the *Nos2* promoter induced by IFN-γ was detected in IEC4.1 cells treated with the Prdm1 siRNA (Fig. 7D). Nevertheless, knockdown of XR_001779380 showed no detectable changes in the assembly of Stat1 to the Prdm1/Pias1 complex in IEC4.1 cells in response to IFN-γ stimulation (Fig. 7E). However, a significant increase of Stat1 assembly to the Swi/Snf complex in response to IFN-γ was detected in IEC4.1 cells treated with the Prdm1 siRNA (Fig. 7F). Taken together, our data support the model that C. parvum infection increases expression of XR_001779380 in intestinal epithelial cells through the TLR4/NF-κB/Cdc42 signaling and epithelial-specific transcription factor Elf3. XR_001779380 modulates Stat1/Swi/Snf-associated chromatin remodeling and primes intestinal epithelial cells for IFN-γ-mediated host antimicrobial gene transcription. However, Prdm1 is highly expressed in neonatal intestinal epithelium and can interact with XR_001779380 to attract Stat1 and form the Prdm1/Pias1/Stat1 complex, thus attenuating Stat1/Swi/Snf-mediated transcription of defense genes in response to IFN-γ (Fig. 7G).

## DISCUSSION

Identification of regulatory lncRNAs in intestinal epithelial cells and characterization of their involvement in epithelial antimicrobial defense may provide new insights into the regulation of gastrointestinal immune homeostasis. The expression of many lncRNAs is highly cell type specific and tightly regulated by lineage-determining proteins or cell type-specific transcription factors (4). Indeed, transcription of XR_001779380 from the *2610027F03Rik* gene is coordinated by epithelial-specific transcription factor Elf3 and by activation of TLR4/Cdc42/NF-κB signaling in intestinal epithelial cells following C. parvum infection or LPS stimulation. It appears that Elf3 and NF-κB subunit p50 are assembled and recruited together to the promoter region of the *2610027F03Rik* gene locus, implicating cross-linking between the Elf3 and NF-κB signaling pathways. Both Elf3 and NF-κB pathways may be downstream signaling for TLR4. Previous studies demonstrated that p21-activated kinase 1 (Pak1), an evolutionarily conserved family of serine/threonine kinases, is an upstream kinase of Elf3 and that Pak1 serine phosphorylation of Elf3 regulates the transforming ability of Elf3 (56, 57). Cdc42 has been implicated in activating Pak1 (58) and, thus, may induce Elf3 nuclear translocation.

Given the fact that TLR/NF-κB signaling is key to mucosal defense, coupled with an emerging role for lncRNAs in immune regulation, we speculate that lncRNAs, such as intestinal epithelial cell-enriched XR_001779380, may be important regulators in gastrointestinal mucosal defense. Specifically, XR_001779380 may act as a coregulator for the interactions between Stat1 and Swi/Snf complex and/or for their chromatin occupancy in intestinal epithelial cells in response to IFN-γ stimulation. Consequently, XR_001779380 primes epithelial cells for IFN-γ-mediated antimicrobial gene transcription through modification of Stat1/Swi/Snf-mediated chromatin remodeling. The Swi/Snf complex is a nucleosome remodeling complex composed of several proteins encoded by the *Swi* and *Snf* genes (i.e., Swi, Brg1, or Brm) (42, 59). The Swi/Snf complex has DNA-stimulated ATPase activity and can destabilize histone-DNA interactions in reconstituted nucleosomes in an ATP-dependent manner (42, 43). The Swi/Snf-mediated chromatin remodeling is a key coregulator to Stat1 signaling to enhance IFN-γ-mediated gene transcription (44–47). It has been demonstrated that the activated phosphorylated form of Stat1 can interact with the Brg1 subunit of the Swi/Snf complex and recruits the complex to IFN-γ-activated sequences of individual genes, eliciting an increase in gene activity (46, 47). Functionally, XR_001779380 may act as a coregulator for the Stat1-Swi/Snf interactions or for their chromatin occupancy in intestinal epithelial cells, as XR_001779380 knockdown significantly attenuated Swi/Snf-associated histone modifications and gene transcription in cells in response to IFN-γ stimulation.

As a key innate defense effector molecule, IFN-γ is mainly synthesized and released from NK cells and other innate lymphoid cells (e.g., groups I and III) at intestinal mucosa (60). Intestinal epithelial cells express various receptors for IFN-γ and can trigger transcription of antimicrobial defense genes upon their ligation (61). Mechanistically, IFN-γ triggers a broad spectrum of cell-intrinsic responses that may target parasite growth by nutrient deprivation and generation of effective defense molecules, such as colony-stimulating factors, C-X-C motif chemokines, and reactive oxygen and nitrogen species (24–26). In our previous studies, we demonstrated that Nos2 is involved in anti-C. parvum defense in intestinal epithelial cells potentially through regulation of nitric oxide production (20). Although it is still not clear how upregulation of certain defense genes would impact epithelial cell anti-C. parvum defense *in vitro*, from an immunological point of view, upregulation of XR_001779380 in intestinal epithelial cells following microbial infection may prime epithelial cells for IFN-γ-mediated antimicrobial gene transcription. This may be relevant to mucosal epithelial antimicrobial defense in general and is of particular importance for epithelial defense to these “mucosal pathogens,” such as *Cryptosporidium*.

While immunocompetent adult individuals are resistant to infection, AIDS patients are highly susceptible to C. parvum infection (12, 13). Similarly, adult mice are resistant to infection; only neonatal mice and adults in immunocompromised strains are susceptible (18, 49). Underlying mechanisms are unclear and have been speculated to be due to immunodeficiency or malnutrition in early life (62, 63). The JAK-STAT signaling pathway appears well developed in intestinal epithelial cells in neonates/infants, but IFN-γ-mediated antimicrobial defense is usually reduced in the cells (64–66). Here, our data from an *ex vivo* enteroid infection model strongly support that Prdm1, which is preferentially expressed in neonatal intestinal epithelial cells (51), may suppress IFN-γ-mediated antimicrobial defense in intestinal epithelial cells, contributing to the higher susceptibility to C. parvum infection in infants or neonates. A high expression level of Prdm1 in the neonatal intestinal epithelium and its absence in adult intestinal epithelium were consistent with a higher infection burden in the neonatal enteroids. Accordingly, adult enteroids showed a stronger transcriptional response to IFN-γ stimulation than neonatal enteroids. Moreover, Prdm1 silencing could restore the response to IFN-γ stimulation in neonatal enteroids to a level comparable to that in the adult enteroids. Of note, PRDM1 has also been demonstrated to be induced in various cell types from AIDS patients during HIV infection (51, 67–69). Given the limitation of the murine models of intestinal cryptosporidiosis (70, 71), the immunological significance for Prdm1 in intestinal cryptosporidiosis warrants future investigations using enteroids derived from humans. Nevertheless, we cannot exclude the potential involvement of insufficient development of adaptive immunity in the neonates.

Prdm1 may suppress IFN-γ-mediated antimicrobial gene transcription in neonatal intestinal epithelial cells through its interaction with Pias1 and XR_001779380. Prdm1 has been demonstrated to interact with Pias1 and modulate Stat1-mediated gene transcription (52, 54). We confirmed the presence of Prdm1 in the Pias1/Stat1 complex precipitated from neonatal intestinal epithelial IEC4.1 cells following IFN-γ stimulation. In addition, we detected physical association of XR_001779380 with Prdm1/Pias1 complex in IEC4.1 cells following IFN-γ stimulation. Knockdown of either Pias1 or Prdm1 promoted the recruitment of Stat1/Swi/Snf complex to the promoter regions of defense gene loci in cells in response to IFN-γ. Therefore, Prdm1 may sequester XR_001779380 and Stat1 to form the Prdm1/Pias1 complex, resulting in inhibition of Stat1/XR_001779380 assembly into the Swi/Snf complex and, consequently, suppression of defense gene transcription in intestinal epithelial cells in response to IFN-γ stimulation. Due to the absence of Prdm1 in adult intestinal epithelial cells, such inhibitory cross-linking is not available in adults and may contribute to associated resistance of infection to *Cryptosporidium*.

Because induction of XR_001779380 was also detected in intestinal epithelium following LPS stimulation, it is plausible to speculate that priming of intestinal epithelial cells by XR_001779380 for IFN-γ-mediated gene transcription may be a general epithelial response to mucosal pathogen infection in the gastrointestinal tract. Nevertheless, it is unclear whether this mechanism is relevant to other mucosal epithelial cells, such as these at the respiratory and urogenital mucosal surfaces. It is also important to know whether XR_001779380 can promote gene transcription in other cells in response to all IFN types, particularly type I and III IFNs. These questions merit future investigations.

## MATERIALS AND METHODS

### C. parvum oocysts.

C. parvum oocysts harvested from calves inoculated with the Iowa strain originally obtained from Harley Moon at the National Animal Disease Center (Ames, IA) were purchased from a commercial source (Bunch Grass Farms, Deary, ID). Oocysts were purified using a modified ether extraction technique and then suspended in phosphate-buffered saline (PBS) and stored at 4°C. For *in vitro* and *in vivo* infection, oocysts were treated with 1% sodium hypochlorite on ice for 20 min and washed 3 times with Dulbecco’s modified Eagle medium (DMEM) culture media. For *ex vivo* infection of cultured enteroids, oocysts were treated with 1% sodium hypochlorite on ice for 20 min and subjected to an excystation solution consisting of 0.75% taurodeoxycholate and 0.25% trypsin for 30 min at 37°C. The excystation rate was calculated as previously described by others (72) and was determined for each new batch of oocysts. These freshly excysted infective sporozoites were collected and added to the cultures for *ex vivo* infection. All parasite preparations were tested using the *Limulus* amebocyte lysate gel formation test as previously described to exclude the possibility of contamination with lipopolysaccharides (73).

### Cell lines, enteroids, and animals.

The neonatal intestinal epithelial cell line (IEC4.1) was a kind gift from Pingchang Yang (McMaster University, Hamilton, Canada). The RAW264.7 mouse macrophage cells were obtained from ATCC. Culture media were supplied with 10% fetal bovine serum (FBS) (Ambion) and antibiotics. Intestinal villus/crypt components from adults or neonatal mice were isolated and cultured as described in our previous studies (32). Briefly, 2.0-cm small intestine tissues above the cecum were collected from adult mice (1.5 cm for neonatal mice) and were opened longitudinally. Collected tissues were then put in 15-ml tubes with 10 ml cold PBS and inverted gently several times to wash the intestinal contents. Obtained tissues were further cut into 1-2 mm pieces and transferred into a 15-ml tube containing 10 ml cold PBS with 2 mmol/liter ethylene‐diamine‐tetraacetic acid, rotated for 30 min at 4°C, and shaken vigorously until it became mostly opaque with dislodged crypt and villus particles. The villus-crypt mix was transferred to 50-ml tubes containing 10 ml cold PBS and filtered through a 70‐μm cell strainer (MidSci; catalog no. 258368‐70). The villus-crypt pellets were collected after centrifugation at 200 × *g* at 4°C for 5 min, washed with 20 ml cold PBS, and centrifuged one more time under the same condition. The pellets were then resuspended with cold PBS-Matrigel media (1:1 ratio in volume) and plated in 35-mm dishes (30 μl mix per spot, 12 spots) or 60-mm dishes (30 spots). All the above steps were carried out in buffers kept on ice. Enteroid culture media (2 ml for 35-mm dishes and 4 ml for 60-mm dishes) were added, and culture medium was changed every other day. The content of enteroid three-dimensional culture medium was epidermal growth factor (EGF), Noggin, R‐spondin 1, B 27 supplement, N2 supplement, gentamicin, 4‐(2‐hydroxyethyl)‐1‐piperazineethanesulfonic acid buffer, and GlutaMAX supplement, with the DMEM-nutrient mixture F‐12 (DMEM-F‐12). For enteroid propagation, EGF, Noggin, R‐spondin 1, and N2 supplement were added immediately before usage of the culture medium.

C57BL/6J mice were originally purchased from the Jackson Laboratory. Mice at the age of 5 days after birth were used as neonates for enteroid isolation or *in vivo* infection. Animals at 6 weeks after birth were used as adults for enteroid isolation. The study was carried out in strict accordance with the recommendations in the Guide for the Care and Use of Laboratory Animals of the National Institutes of Health under the Assurance of Compliance number A3348-01. All animal experiments were done in accordance with procedures (protocol number 0959) approved by the Institutional Animal Care and Use Committee of Creighton University.

### Infection models and assays.

For *in vitro* infection using IEC4.1 cells, infection was done in culture medium (DMEM-F-12 with 100 U/ml penicillin and 100 μg/ml streptomycin) containing viable C. parvum oocysts after treatment with 1% sodium hypochlorite (oocysts with host cells in a 1:1 ratio). Cells were then cultured for 4 h at 37°C for attachment and invasion by the parasites. After extensive washing with DMEM-F-12 medium three times to remove free parasites. Cells were cultured for additional time periods as described. A model of C. parvum infection using enteroids was employed as previously described (32). The crypt/villus units immediately after isolation were exposed to freshly excysted C. parvum sporozoites for 10 min at 37°C and then for an additional 2 h on ice, followed by culture at 37°C in the enteroid three-dimensional culture medium at various time periods as described above. The neonatal murine infection model of intestinal cryptosporidiosis was used for *in vivo* experiments (29, 30, 74). Neonates (5 days after birth) received C. parvum oocysts by oral gavage (10^5^ oocysts per mouse) to develop intestinal cryptosporidiosis. Mice that received PBS by oral gavage were used as control. At 24, 48, and 72 h after C. parvum oocysts or PBS administration, animals were sacrificed, and ileum intestine tissues were collected. At least five animals from each group were sacrificed, and ileum epithelium tissues were obtained for biochemical analyses. qRT-PCR, immunofluorescence microscopy, and immunohistochemistry were used to assay C. parvum infection as previously reported (72, 75, 76). The intestinal tissues were collected and parasite burden was performed by counting all intracellular parasite stages as previously reported (75). For immunohistochemistry, tissue slides were first incubated with the primary antibodies, followed by the secondary antibody (Molecular Probes), and visualized according to the standard approach.

### qRT-PCR.

For quantitative analysis of RNA and C. parvum RNA expression, comparative qRT-PCR was performed as previously reported (32, 75–77), using the SYBR green PCR master mix (Applied Biosystems, Carlsbad, CA, USA). Briefly, total RNA was isolated and possible remaining DNA was removed using TRI reagent and treated with DNA-free kit (Ambion, MA, USA). qRT-PCR was then performed using 25 ng of template cDNA from reverse transcription for each RNA gene of interest. The expression level of each RNA was calculated using the threshold cycle (ΔΔ*C_T_*) method and normalized to glyceraldehyde-3-phosphate dehydrogenase (*Gapdh*). All sequences of PCR primers are listed in Table S1 in the supplemental material.

10.1128/mBio.02127-21.8 TABLE S1 List of siRNAs, antibodies, and primers used for real-time PCR, generating constructs, and probe sequences for ChIRP analysis. Download Table S1, XLSX file, 0.02 MB. Copyright © 2021 Gong et al. 2021 Gong et al. https://creativecommons.org/licenses/by/4.0/ This content is distributed under the terms of the Creative Commons Attribution 4.0 International license.

### Transfections with siRNAs and plasmids.

Custom-designed RNA oligonucleotides against XR_001779380 and two scrambled RNAs (as the siRNA control) were synthesized by IDT (Integrated DNA Technologies, Coralville, IA) and transfected into cells with Lipofectamine RNAiMAX according to the manufacturer’s protocol (Invitrogen). siRNAs to Elf3, Snf5, and Prdm1 were purchased from the Santa Cruz Biotechnology. Sequences of siRNAs are listed in Table S1. siRNAs were transfected into IEC4.1 cells with Lipofectamine RNAiMAX. For siRNA transfection into enteroids, the electroporation approach was used with the Neon transfection system (Thermo Fisher Scientific, Waltham, MA; electroporation parameter, 1600 V, 10 ms, pulse 3). The sequence of XR_001779380 was cloned into the pTarget vector according to the manufacturer’s protocol. XR_001779380-pTarget was transfected to cells with Lipofectamine 2000 (Invitrogen). The mouse Elf3 CRISPR/Cas9 knockout and the control plasmids were purchased from Santa Cruz Biotechnology. The pKR5-Cdc42(17N)-Myc construct (a dominant negative form) (78) was a gift from A. Hall (University College London, London, United Kingdom), and the TLR4-DN was kindly provided by M. F. Smith (University of Virginia, Charlottesville, VA).

### Nuclear extracts and Western blotting.

Nuclear extracts were isolated using the standard approach (73, 79, 80). Briefly, cells were grown to 80% confluence and then exposed to C. parvum for various times. Cells were treated with EDTA (Sigma) and washed with PBS, and the cell pellet was resuspended in 1 ml of cold buffer A (10 mM HEPES, 1.5 mM MgCl_2_, 10 mM KCl, and 1 mM dithiothreitol [DTT]). Nuclear pellets were isolated from the whole-cell protein by centrifugation at 14,000 rpm for 1 min at 4°C and resuspended in cold buffer B (20 mM HEPES, 1.5 mM MgCl_2_, 25% glycerol, 420 mM NaCl, 0.2 mM EDTA, and 1 mM DTT) with vigorous agitation in the cold room for 30 min. The supernatant containing nuclear proteins was collected after centrifugation at 14,000 rpm for 5 min at 4°C. Protein concentration of each nuclear extract or whole-cell lysate was determined and subsequently analyzed by Western blotting. The following antibodies were used for blotting (details in Table S1): anti-Brg1 (Santa Cruz), anti-H3K36me3 (Abcam), anti-H3K4me3 (Abcam), anti-Stat1 (Cell Signaling), anti-pStat1 (Cell Signaling), anti-Pias1 (Santa Cruz), anti-Snf5 (Santa Cruz), anti-Baf170 (Cell Signaling), anti-p50 (Abcam), anti-Elf3 (Santa Cruz), anti-Cdc42 (Fisher Scientific), anti-Actin (Sigma), and anti-Prdm1 (Cell Signaling).

### RNA stability.

RNA stability assay was performed by qRT-PCR as previously reported (76, 81). Briefly, cells were treated with actinomycin D (10 μg/ml, Fisher) to block transcription, and RNAs were isolated at various time points after actinomycin D treatment. qRT-PCR was then performed using 25 ng of template cDNA for each mRNA gene of interest. Each sample was run in triplicate. The relative abundance of each mRNA was calculated using the ΔΔ*C_T_* method and normalized to Gapdh. The relative amount of mRNA at 0 h following actinomycin D treatment was arbitrarily set to 1. Curve fittings of the resultant data were performed using Microsoft Excel and the half-lives of the RNAs calculated.

### IP and co-IP.

Cells were lysed with the lysis buffer (20 mM Tris-HCl, pH 8.0, 150 mM NaCl, 1% NP-40, 20 μM MG132, 1 mM phenylmethylsulfonyl fluoride [PMSF], 10 μg/ml leupeptin, and 2 μg/ml pepstatin). A total of 500 μg of lysate protein was incubated with the primary antibodies at 4°C overnight to immunoprecipitate the protein complexes. Immune complexes were then collected by direct binding to protein A-Sepharose. The immunoprecipitates were then blotted with the corresponding antibody against the protein as indicated. Anti-Prdm1 (Cell Signaling), anti-Baf170 (Cell Signaling), anti-pStat1 (Cell Signaling), anti-Stat1 (Cell Signaling), anti-Snf5 (Santa Cruz), anti-Pias1 (Santa Cruz), anti-p50 (Cell Signaling), anti-IgG (Santa Cruz), and anti-Elf3 (Santa Cruz) were used for IP/co-IP analysis.

### RIP, ChIP, and ChIRP analyses.

The formaldehyde cross-linking RIP was performed as described (82). Briefly, cells in culture were first treated with 0.05 M EDTA/PBS, washed once with culture medium containing 10% FBS, washed twice with 10 ml PBS, and resuspended in 10 ml of PBS. Formaldehyde (37% stock solution) was then added to a final concentration of 0.3% (vol/vol) and incubated at room temperature for 10 min with slow mixing. Cross-linking reactions were quenched by the addition of glycine (pH 7.0) to a final concentration of 0.25 M followed by incubation at room temperature for 5 min. The cells then harvested by centrifugation using a clinical centrifuge at 3,000 rpm (237 × *g*) for 4 min followed by two washes with ice-cold PBS. Cell pellets were resuspended in 1 ml of lysis buffer (10 mM Tris-HCl pH 7.4, 10 mM NaCl, 3 mM MgCl_2_, 0.5% NP-40, and cocktail protease inhibitor and RNase inhibitor, 100 units). After a 10-min incubation on ice, nuclei were collected by centrifugation (500 × *g*, 5 min) and washed with lysis buffer devoid of NP-40. After centrifugation, the pellets were resuspended in 100 μl nuclei lysis buffer (10 mM Tris-HCl, pH 7.4, 400 mM NaCl, 1 mM EDTA, 1 mM DTT, and cocktail protease inhibitor plus RNase inhibitor, 10 units) and mixed thoroughly for 30 min at 4°C. The nuclei lysates were diluted 5-fold in WCE buffer (20 mM HEPES, pH 7.4, 0.2 M NaCl, 0.5% Triton X-100, 10% glycerol, 1 mM EDTA, 1 mM EGTA, 10 mM β-glycerophosphate, 2 mM Na3VO4, 1 mM NaF, 1 mM DTT, and cocktail protease inhibitor plus RNase inhibitor). Solubilization of cross-linked complexes was done by mechanical sonication by three rounds of sonication, 20 s each, in a Microson XL2007 ultrasonic homogenizer with a microprobe at an amplitude setting of 7 (output, 8 to 9 W). Insoluble materials were removed by microcentrifugation at 14,000 rpm (16,000 × *g*) for 20 min at 4°C. Preclearing lysates with 20 μl of PBS-washed Magna ChIP protein A+G magnetic beads (Millipore, Massachusetts). The precleared lysate (250 μl) was then diluted with WCE buffer (250 μl), mixed with the specific antibody-coated beads, and incubated with rotation at 4°C for 4 h, followed by 4 times washing with WCE buffer containing protease and RNase inhibitors. The collected immunoprecipitated RNP complexes and input were digested in RNA PK buffer, pH 7.0 (100 mM NaCl, 10 mM Tris-HCl, pH 7.0, 1 mM EDTA, and 0.5% SDS), with addition of 10 μg proteinase K and incubated at 50°C for 45 min with end-to-end shaking at 400 rpm. Formaldehyde cross-links were reversed by incubation at 65°C with rotation for 4 h. RNA was extracted from these samples using TRIzol reagent according to the manufacturer’s protocol (Invitrogen Corp.) and treated with DNA-free DNase removal kit according to the manufacturer’s protocol (Ambion Inc., Austin, TX). The presence of RNA was measured by quantitative, strand-specific qRT-PCR using the iCycler iQ real-time detection system (Bio-Rad). Gene-specific PCR primer pairs are listed in Table S1. ChIP assay was performed as previously reported (8, 10, 11) using the ChIP Assay Kit (Millipore Sigma). The following antibodies (details in Table S1) were used for RIP or ChIP analysis: anti-H3K36me3 (Abcam), anti-H3K4me3 (Abcam), anti-Stat1 (Cell Signaling), anti-IgG (Santa Cruz), and anti-Brg1 (Santa Cruz).

ChIRP analysis was performed as previously reported (83). Briefly, glutaraldehyde cross-linked for chromatin isolation and a pool of tiling oligonucleotide probes with affinity specific to the XR_001779380 sequence were used. The sequences for each probe are listed in Table S1. The DNA sequences of the chromatin immunoprecipitates were confirmed by qRT-PCR using the same primer sets covering the gene promoter regions of interest as for ChIP analysis. A pool of scrambled oligonucleotide probes for *LacZ* (Table S1) was used as controls.

### Statistical analysis.

Statistical analysis was performed using GraphPad Prism 5 (GraphPad Software). All values are given as mean ± standard deviation (SD). Means of groups were from at least three independent experiments and compared with Student's *t* test (unpaired) or the analysis of variance (ANOVA) test when appropriate. *P* values of <0.05 were considered statistically significant.

## References

[1] ENCODE Project Consortium. 2007. Identification and analysis of functional elements in 1% of the human genome by the ENCODE pilot project. Nature 447:799–816. doi:10.1038/nature05874. 17571346 PMC2212820

[2] The FANTOM Consortium. 2005. The transcriptional landscape of the mammalian genome. Science 309:1559–1563. doi:10.1126/science.1112014. 16141072

[3] Hu W, Alvarez-Dominguez JR, Lodish HF. 2012. Regulation of mammalian cell differentiation by long non-coding RNAs. EMBO Rep 13:971–983. doi:10.1038/embor.2012.145. 23070366 PMC3492712

[4] Rinn JL, Chang HY. 2012. Genome regulation by long noncoding RNAs. Annu Rev Biochem 81:145–166. doi:10.1146/annurev-biochem-051410-092902. 22663078 PMC3858397

[5] Mathy NW, Chen XM. 2017. LincRNAs and their transcriptional control of inflammatory responses. J Biol Chem 292:12375–12382. doi:10.1074/jbc.R116.760884. 28615453 PMC5535013

[6] Jiang C, Li Y, Zhao Z, Lu J, Chen H, Ding N, Wang G, Xu J, Li X. 2016. Identifying and functionally characterizing tissue-specific and ubiquitously expressed human lincRNAs. Oncotarget 7:7120–7133. doi:10.18632/oncotarget.6859. 26760768 PMC4872773

[7] Peng X, Gralinski L, Armour CD, Ferris MT, Thomas MJ, Proll S, Bradel-Tretheway BG, Korth MJ, Castle JC, Biery MC, Bouzek HK, Haynor DR, Frieman MB, Heise M, Raymond CK, Baric RS, Katze MG. 2010. Unique signatures of long noncoding RNA expression in response to virus infection and altered innate immune signaling. mBio 1:e00206-10. doi:10.1128/mBio.00206-10. 20978541 PMC2962437

[8] Li M, Gong AY, Zhang X, Wang Y, Mathy NW, Martins GA, Strauss-Soukup JK, Chen XM. 2018. Induction of a long non-coding RNA transcript, NR_045064, promotes defense gene transcription and facilitates intestinal epithelial cell responses against *Cryptosporidium* infection. J Immunol 201:3630–3640. doi:10.4049/jimmunol.1800566. 30446564 PMC6289618

[9] Guttman M, Amit I, Garber M, French C, Lin MF, Feldser D, Huarte M, Zuk O, Carey BW, Cassady JP, Cabili MN, Jaenisch R, Mikkelsen TS, Jacks T, Hacohen N, Bernstein BE, Kellis M, Regev A, Rinn JL, Lander ES. 2009. Chromatin signature reveals over a thousand highly conserved large non-coding RNAs in mammals. Nature 458:223–227. doi:10.1038/nature07672. 19182780 PMC2754849

[10] Hu G, Gong AY, Wang Y, Ma S, Chen X, Chen J, Su CJ, Shibata A, Strauss-Soukup JK, Drescher KM, Chen XM. 2016. LincRNA-Cox2 promotes late inflammatory gene transcription in macrophages through modulating SWI/SNF-mediated chromatin remodeling. J Immunol 196:2799–2808. doi:10.4049/jimmunol.1502146. 26880762 PMC4779692

[11] Tong Q, Gong AY, Zhang X, Lin C, Ma S, Chen J, Hu G, Chen XM. 2016. LincRNA-Cox2 modulates TNFα-induced transcription of *Il12b* gene in intestinal epithelial cells through regulation of Mi-2/NuRD-mediated epigenetic histone modifications. FASEB J 30:1187–1197. doi:10.1096/fj.15-279166. 26578685 PMC4750408

[12] Striepen B. 2013. Parasitic infections: time to tackle cryptosporidiosis. Nature 503:189–191. doi:10.1038/503189a. 24236315

[13] Checkley W, White AC, Jaganath D, Arrowood MJ, Chalmers RM, Chen X-M, Fayer R, Griffiths JK, Guerrant RL, Hedstrom L, Huston CD, Kotloff KL, Kang G, Mead JR, Miller M, Petri WA, Priest JW, Roos DS, Striepen B, Thompson RCA, Ward HD, Van Voorhis WA, Xiao L, Zhu G, Houpt ER. 2015. Cryptosporidiosis: global burden, novel diagnostics, therapeutics and vaccine targets for cryptosporidium. Lancet Infect Dis 15:85–94. doi:10.1016/S1473-3099(14)70772-8. 25278220 PMC4401121

[14] Kotloff KL, Nataro JP, Blackwelder WC, Nasrin D, Farag TH, Panchalingam S, Wu Y, Sow SO, Sur D, Breiman RF, Faruque AS, Zaidi AK, Saha D, Alonso PL, Tamboura B, Sanogo D, Onwuchekwa U, Manna B, Ramamurthy T, Kanungo S, Ochieng JB, Omore R, Oundo JO, Hossain A, Das SK, Ahmed S, Qureshi S, Quadri F, Adegbola RA, Antonio M, Hossain MJ, Akinsola A, Mandomando I, Nhampossa T, Acácio S, Biswas K, O'Reilly CE, Mintz ED, Berkeley LY, Muhsen K, Sommerfelt H, Robins-Browne RM, Levine MM. 2013. Burden and aetiology of diarrhoeal disease in infants and young children in developing countries (the Global Enteric Multicenter Study, GEMS): a prospective, case-control study. Lancet 382:209–222. doi:10.1016/S0140-6736(13)60844-2. 23680352

[15] Khalil IA, Troeger C, Rao PC, Blacker BF, Rown A, Brewer TG, Colombara DV, Hostos ELD, Engmann C, Guerrant RL, Haque R, Houpt ER, Kang G, Korpe PS, Kotloff KL, Lima AAM, Petri WA, Jr., Platts-Mills JA, Shoultz DA, Forouzanfar MH, Hay SI, Reiner RC, Jr., Mokdad AH. 2018. Morbidity, mortality, and long-term consequences associated with diarrhoea from *Cryptosporidium* infection in children younger than 5 years: a meta-analyses study. Lancet Glob Health 6:e758–e768. doi:10.1016/S2214-109X(18)30283-3. 29903377 PMC6005120

[16] Putignani L, Menichella D. 2010. Global distribution, public health and clinical impact of the protozoan pathogen *Cryptosporidium*. Interdiscip Perspect Infect Dis 2010:753512. doi:10.1155/2010/753512. 20706669 PMC2913630

[17] Pierce KK, Kirkpatrick BD. 2009. Update on human infections caused by intestinal protozoa. Curr Opin Gastroenterol 25:12–17. doi:10.1097/mog.0b013e32831da7dd. 19119509

[18] Chen XM, Keithly JS, Paya CV, LaRusso NF. 2002. Cryptosporidiosis. N Engl J Med 346:1723–1731. doi:10.1056/NEJMra013170. 12037153

[19] Ming ZP, Zhou R, Chen XM. 2017. Regulation of host epithelial responses to *Cryptosporidium* infection by microRNAs. Parasite Immunol 39:e12408. doi:10.1111/pim.12408. 27977858

[20] Zhou R, Gong AY, Eischeid AN, Chen XM. 2012. miR-27b targets KSRP to coordinate TLR4-mediated epithelial defense against *Cryptosporidium parvum* infection. PLoS Pathog 8:e1002702. doi:10.1371/journal.ppat.1002702. 22615562 PMC3355088

[21] Laurent F, Kagnoff MF, Savidge TC, Naciri M, Eckmann L. 1998. Human intestinal epithelial cells respond to *Cryptosporidium parvum* infection with increased prostaglandin H synthase 2 expression and prostaglandin E2 and F2alpha production. Infect Immun 66:1787–1790. doi:10.1128/IAI.66.4.1787-1790.1998. 9529115 PMC108122

[22] Pollok RC, Farthing MJ, Bajaj-Elliott M, Sanderson IR, McDonald V. 2001. Interferon gamma induces enterocyte resistance against infection by the intracellular pathogen *Cryptosporidium parvum*. Gastroenterology 120:99–107. doi:10.1053/gast.2001.20907. 11208718

[23] Pantenburg B, Castellanos-Gonzalez A, Dann SM, Connelly RL, Lewis DE, Ward HD, White AC. Jr., 2010. Human CD8(+) T cells clear *Cryptosporidium parvum* from infected intestinal epithelial cells. Am J Trop Med Hyg 82:600–607. doi:10.4269/ajtmh.2010.09-0590. 20348507 PMC2844566

[24] Bedi B, McNair NN, Förster I, Mead JR. 2015. IL-18 cytokine levels modulate innate immune responses and cryptosporidiosis in mice. J Eukaryot Microbiol 62:44–50. doi:10.1111/jeu.12164. 25155632

[25] Choudhry N, Petry F, van Rooijen N, McDonald V. 2012. A protective role for interleukin 18 in interferon γ-mediated innate immunity to *Cryptosporidium parvum* that is independent of natural killer cells. J Infect Dis 206:117–124. doi:10.1093/infdis/jis300. 22517912

[26] Barakat FM, McDonald V, Di Santo JP, Korbel DS. 2009. Roles for NK cells and an NK cell-independent source of intestinal gamma interferon for innate immunity to *Cryptosporidium parvum* infection. Infect Immun 77:5044–5049. doi:10.1128/IAI.00377-09. 19687195 PMC2772539

[27] Church DM, Schneider VA, Graves T, Auger K, Cunningham F, Bouk N, Chen H-C, Agarwala R, McLaren WM, Ritchie GRS, Albracht D, Kremitzki M, Rock S, Kotkiewicz H, Kremitzki C, Wollam A, Trani L, Fulton L, Fulton R, Matthews L, Whitehead S, Chow W, Torrance J, Dunn M, Harden G, Threadgold G, Wood J, Collins J, Heath P, Griffiths G, Pelan S, Grafham D, Eichler EE, Weinstock G, Mardis ER, Wilson RK, Howe K, Flicek P, Hubbard T. 2011. Modernizing reference genome assemblies. PLoS Biol 9:e1001091. doi:10.1371/journal.pbio.1001091. 21750661 PMC3130012

[28] Li XC, Jevnikar AM, Grant DR. 1997. Expression of functional ICAM-1 and VCAM-1 adhesion molecules by an immortalized epithelial cell clone derived from the small intestine. Cell Immunol 175:58–66. doi:10.1006/cimm.1996.1050. 9015189

[29] Lacroix S, Mancassola R, Naciri M, Laurent F. 2001. *Cryptosporidium parvum*-specific mucosal immune response in C57BL/6 neonatal and gamma interferon-deficient mice: role of tumor necrosis factor alpha in protection. Infect Immun 69:1635–1642. doi:10.1128/IAI.69.3.1635-1642.2001. 11179338 PMC98067

[30] Kapel N, Benhamou Y, Buraud M, Magne D, Opolon P, Gobert J-G, Magne D. 1996. Kinetics of mucosal ileal gamma-interferon response during cryptosporidiosis in immunocompetent neonatal mice. Parasitol Res 82:664–667. doi:10.1007/s004360050182. 8897498

[31] Deng M, Lancto CA, Abrahamsen MS. 2004. *Cryptosporidium parvum* regulation of human epithelial cell gene expression. Int J Parasitol 34:73–82. doi:10.1016/j.ijpara.2003.10.001. 14711592

[32] Zhang XT, Gong AY, Wang Y, Chen X, Lim SYS, Dolata CE, Chen XM. 2016. *Cryptosporidium parvum* infection attenuates the *ex vivo* propagation of murine intestinal enteroids. Physiol Rep 4:e13060. doi:10.14814/phy2.13060. 28039407 PMC5210379

[33] Ma S, Ming ZP, Gong AY, Wang Y, Chen X, Hu G, Zhou R, Shibata A, Swanson PC, Chen XM. 2017. A long non-coding RNA, LincRNA-Tnfaip3, acts as a co-regulator of NF-κB to modulate inflammatory gene transcription in mouse macrophages. FASEB J 31:1215–1225. doi:10.1096/fj.201601056R. 27979905 PMC6191062

[34] Chen XM, O'Hara SP, Nelson JB, Splinter PL, Small AJ, Tietz PS, Limper AH, LaRusso NF. 2005. Multiple TLRs are expressed in human cholangiocytes and mediate host epithelial defense responses to *Cryptosporidium parvum* via activation of NF-kappaB. J Immunol 175:7447–7756. doi:10.4049/jimmunol.175.11.7447. 16301652

[35] Oikawa T, Yamada T. 2003. Molecular biology of the Ets family of transcription factor. Gene 303:11–34. doi:10.1016/s0378-1119(02)01156-3. 12559563

[36] Silberg DG, Swain GP, Suh ER, Traber PG. 2000. Cdx1 and Cdx2 expression during intestinal development. Gastroenterology 119:961–971. doi:10.1053/gast.2000.18142. 11040183

[37] Kast C, Wang M, Whiteway M. 2003. The ERK/MAPK pathway regulates the activity of the human tissue factor pathway inhibitor-2 promoter. J Biol Chem 278:6787–6794. doi:10.1074/jbc.M210935200. 12446683

[38] Musikacharoen T, Matsuguchi T, Kikuchi T, Yoshikai Y. 2001. NF-kappa B and STAT5 play important roles in the regulation of mouse Toll-like receptor 2 gene expression. J Immunol 166:4516–4524. doi:10.4049/jimmunol.166.7.4516. 11254708

[39] Kishore N, Sommers C, Mathialagan S, Guzova J, Yao M, Hauser S, Huynh K, Bonar S, Mielke C, Albee L, Weier R, Graneto M, Hanau C, Perry T, Tripp CS. 2003. A selective IKK-2 inhibitor blocks NF-kappa B-dependent gene expression in interleukin-1 beta-stimulated synovial fibroblasts. J Biol Chem 278:32861–32871. doi:10.1074/jbc.M211439200. 12813046

[40] Chen XM, Huang BQ, Splinter PL, Orth JD, Billadeau DD, McNiven MA, LaRusso NF. 2004. Cdc42 and the actin-related protein/neural Wiskott-Aldrich syndrome protein network mediate cellular invasion by *Cryptosporidium parvum*. Infect Immun 72:3011–3021. doi:10.1128/IAI.72.5.3011-3021.2004. 15102814 PMC387898

[41] Majoros A, Platanitis E, Kernbauer-Hölzl E, Rosebrock F, Müller M, Decker T. 2017. Canonical and non-canonical aspects of JAK-STAT signaling: lessons from interferons for cytokine responses. Front Immunol 8:29. doi:10.3389/fimmu.2017.00029. 28184222 PMC5266721

[42] Euskirchen GM, Auerbach RK, Davidov E, Gianoulis TA, Zhong G, Rozowsky J, Bhardwaj N, Gerstein MB, Snyder M. 2011. Diverse roles and interactions of the SWI/SNF chromatin remodeling complex revealed using global approaches. PLoS Genet 7:e1002008. doi:10.1371/journal.pgen.1002008. 21408204 PMC3048368

[43] Bouazoune K, Miranda TB, Jones PA, Kingston RE. 2009. Analysis of individual remodeled nucleosomes reveals decreased histone-DNA contacts created by hSWI/SNF. Nucleic Acids Res 37:5279–5294. doi:10.1093/nar/gkp524. 19567737 PMC2760786

[44] Zhang Y, Cheng MB, Zhang YJ, Zhong X, Dai H, Yan L, Wu NH, Zhang Y, Shen YF. 2010. A switch from hBrm to Brg1 at IFNγ-activated sequences mediates the activation of human genes. Cell Res 20:1345–1360. doi:10.1038/cr.2010.155. 21079652

[45] Yan Z, Cui K, Murray DM, Ling C, Xue Y, Gerstein A, Parsons R, Zhao K, Wang W. 2005. PBAF chromatin-remodeling complex requires a novel specificity subunit, BAF200, to regulate expression of selective interferon-responsive genes. Genes Dev 19:1662–1667. doi:10.1101/gad.1323805. 15985610 PMC1176002

[46] Liu H, Kang H, Liu R, Chen X, Zhao K. 2002. Maximal induction of a subset of interferon target genes requires the chromatin-remodeling activity of the BAF complex. Mol Cell Biol 22:6471–6479. doi:10.1128/MCB.22.18.6471-6479.2002. 12192045 PMC135632

[47] Pattenden SG, Klose R, Karaskov E, Bremner R. 2002. Interferon-gamma-induced chromatin remodeling at the CIITA locus is BRG1 dependent. EMBO J 21:1978–1986. doi:10.1093/emboj/21.8.1978. 11953317 PMC125964

[48] Prensner JR, Iyer MK, Sahu A, Asangani IA, Cao Q, Patel L, Vergara IA, Davicioni E, Erho N, Ghadessi M, Jenkins RB, Triche TJ, Malik R, Bedenis R, McGregor N, Ma T, Chen W, Han S, Jing X, Cao X, Wang X, Chandler B, Yan W, Siddiqui J, Kunju LP, Dhanasekaran SM, Pienta KJ, Feng FY, Chinnaiyan AM. 2013. The long noncoding RNA SChLAP1 promotes aggressive prostate cancer and antagonizes the SWI/SNF complex. Nat Genet 45:1392–1398. doi:10.1038/ng.2771. 24076601 PMC3812362

[49] Pantenburg B, Dann SM, Wang HC, Robinson P, Castellanos-Gonzalez A, Lewis DE, White AC. Jr., 2008. Intestinal immune response to human *Cryptosporidium spp*. infection. Infect Immun 76:23–29. doi:10.1128/IAI.00960-07. 17967863 PMC2223661

[50] John SA, Garrett-Sinha LA. 2009. Blimp1: a conserved transcriptional repressor critical for differentiation of many tissues. Exp Cell Res 315:1077–1084. doi:10.1016/j.yexcr.2008.11.015. 19073176

[51] Harper J, Mould A, Andrews RM, Bikoff EK, Robertson EJ. 2011. The transcriptional repressor Blimp1/Prdm1 regulates postnatal reprogramming of intestinal enterocytes. Proc Natl Acad Sci USA 108:10585–10590. doi:10.1073/pnas.1105852108. 21670299 PMC3127883

[52] Kim SJ. 2015. Immunological function of Blimp-1 in dendritic cells and relevance to autoimmune diseases. Immunol Res 63:113–120. doi:10.1007/s12026-015-8694-5. 26376898 PMC4651792

[53] Smith MA, Maurin M, Cho HI, Becknell B, Freud AG, Yu J, Wei S, Djeu J, Celis E, Caligiuri MA, Wright KL. 2010. PRDM1/Blimp-1 controls effector cytokine production in human NK cells. J Immunol 185:6058–6067. doi:10.4049/jimmunol.1001682. 20944005 PMC3864810

[54] Ying HY, Su ST, Hsu PH, Chang CC, Lin IY, Tseng YH, Tsai MD, Shih HM, Lin KI. 2012. SUMOylation of Blimp-1 is critical for plasma cell differentiation. EMBO Rep 13:631–637. doi:10.1038/embor.2012.60. 22555612 PMC3388782

[55] Zazzo ED, Rosa CD, Abbondanza C, Moncharmont B. 2013. PRDM proteins: molecular mechanisms in signal transduction and transcriptional regulation. Biology (Basel) 2:107–141. doi:10.3390/biology2010107. 24832654 PMC4009873

[56] Manavathi B, Rayala SK, Kumar R. 2007. Phosphorylation-dependent regulation of stability and transforming potential of ETS transcriptional factor ESE-1 by p21-activated kinase 1. J Biol Chem 282:19820–19830. doi:10.1074/jbc.M702309200. 17491012

[57] Oliver JR, Kushwah R, Jim Hu J. 2012. Multiple roles of the epithelium-specific ETS transcription factor, ESE-1, in development and disease. Lab Invest 92:320–330. doi:10.1038/labinvest.2011.186. 22157719

[58] Kong L, Ge BX. 2008. MyD88-independent activation of a novel actin-Cdc42/Rac pathway is required for Toll-like receptor-stimulated phagocytosis. Cell Res 18:745–755. doi:10.1038/cr.2008.65. 18542102

[59] Fan HY, Trotter KW, Archer TK, Kingston RE. 2005. Swapping function of two chromatin remodeling complexes. Mol Cell 17:805–815. doi:10.1016/j.molcel.2005.02.024. 15780937

[60] Sedda S, Marafini I, Figliuzzi MM, Pallone F, Monteleone G. 2014. An overview of the role of innate lymphoid cells in gut infections and inflammation. Mediators Inflamm 2014:235460. doi:10.1155/2014/235460. 25061260 PMC4100280

[61] Beaurepaire C, Smyth D, McKay DM. 2009. Interferon-gamma regulation of intestinal epithelial permeability. J Interferon Cytokine Res 29:133–144. doi:10.1089/jir.2008.0057. 19196071

[62] Liu J, Bolick DT, Kolling GL, Fu Z, Guerrant RL. 2016. Protein malnutrition impairs intestinal epithelial cell turnover, a potential mechanism of increased cryptosporidiosis in a murine model. Infect Immun 84:3542–3549. doi:10.1128/IAI.00705-16. 27736783 PMC5116730

[63] Costa LB, JohnBull EA, Reeves JT, Sevilleja JE, Freire RS, Hoffman PS, Lima AA, Oriá RB, Roche JK, Guerrant RL, Warren CA. 2011. *Cryptosporidium*-malnutrition interactions: mucosal disruption, cytokines, and TLR signaling in a weaned murine model. J Parasitol 97:1113–1120. doi:10.1645/GE-2848.1. 21711105 PMC3247658

[64] Kollmann TR, Levy O, Montgomery RR, Goriely S. 2012. Innate immune function by Toll-like receptors: distinct responses in newborns and the elderly. Immunity 37:771–783. doi:10.1016/j.immuni.2012.10.014. 23159225 PMC3538030

[65] Chang BA, Huang Q, Quan J, Chau V, Ladd M, Kwan E, McFadden DE, Lacaze-Masmonteil T, Miller SP, Lavoie PM. 2011. Early inflammation in the absence of overt infection in preterm neonates exposed to intensive care. Cytokine 56:621–626. doi:10.1016/j.cyto.2011.08.028. 21940177 PMC4494824

[66] Danis B, George TC, Goriely S, Dutta B, Renneson J, Gatto L, Fitzgerald-Bocarsly P, Marchant A, Goldman M, Willems F, De Wit D. 2008. Interferon regulatory factor 7-mediated responses are defective in cord blood plasmacytoid dendritic cells. Eur J Immunol 38:507–517. doi:10.1002/eji.200737760. 18200500

[67] Seddiki N, Phetsouphanh C, Swaminathan S, Xu Y, Rao S, Li J, Sutcliffe EL, Denyer G, Finlayson R, Gelgor L, Cooper DA, Zaunders J, Kelleher AD. 2013. The microRNA-9/B-lymphocyte-induced maturation protein-1/IL-2 axis is differentially regulated in progressive HIV infection. Eur J Immunol 43:510–520. doi:10.1002/eji.201242695. 23129528

[68] Shankar EM, Che KF, Messmer D, Lifson JD, Larsson M. 2011. Expression of a broad array of negative costimulatory molecules and Blimp-1 in T cells following priming by HIV-1 pulsed dendritic cells. Mol Med 17:229–240. doi:10.2119/molmed.2010.00175. 21103670 PMC3060986

[69] de Masson A, Kirilovsky A, Zoorob R, Avettand-Fenoel V, Morin V, Oudin A, Descours B, Rouzioux C, Autran B. 2014. Blimp-1 overexpression is associated with low HIV-1 reservoir and transcription levels in central memory CD4+ T cells from elite controllers. AIDS 28:1567–1577. doi:10.1097/QAD.0000000000000295. 24804861

[70] Leitch GJ, He Q. 1999. Reactive nitrogen and oxygen species ameliorate experimental cryptosporidiosis in the neonatal BALB/c mouse model. Infect Immun 67:5885–5891. doi:10.1128/IAI.67.11.5885-5891.1999. 10531244 PMC96970

[71] Favennec L. 1997. Physiopathologic and therapeutic studies in *in vitro* and *in vivo* models of *Cryptosporidium parvum* infection. J Eukaryot Microbiol 44:69S–70S. doi:10.1111/j.1550-7408.1997.tb05785.x. 9508450

[72] Chen XM, Levine SA, Splinter PL, Tietz PS, Ganong AL, Jobin C, Gores GJ, Paya CV, LaRusso NF. 2001. *Cryptosporidium parvum* activates nuclear factor kappaB in biliary epithelia preventing epithelial cell apoptosis. Gastroenterology 120:1774–1783. doi:10.1053/gast.2001.24850. 11375958

[73] Wang Y, Gong AY, Ma S, Chen X, Li Y, Su CJ, Norall D, Chen J, Strauss-Soukup JK, Chen XM. 2017. Delivery of parasite RNA transcripts into infected epithelial cells during *Cryptosporidium* infection and its potential impact on host gene transcription. J Infect Dis 215:636–643. doi:10.1093/infdis/jiw607. 28007919 PMC5388288

[74] Novak SM, Sterling CR. 1991. Susceptibility dynamics in neonatal BALB/c mice infected with *Cryptosporidium parvum*. J Protozool 38:103S–104S. 1818125

[75] Sasahara T, Maruyama H, Aoki M, Kikuno R, Sekiguchi T, Takahashi A, Satoh Y, Kitasato H, Takayama Y, Inoue M. 2003. Apoptosis of intestinal crypt epithelium after *Cryptosporidium parvum* infection. J Infect Chemother 9:278–281. doi:10.1007/s10156-003-0259-1. 14513402

[76] Zhou R, Hu G, Liu J, Gong AY, Drescher KM, Chen XM. 2009. NF-kappaB p65-dependent transactivation of miRNA genes following *Cryptosporidium parvum* infection stimulates epithelial cell immune responses. PLoS Pathog 5:e1000681. doi:10.1371/journal.ppat.1000681. 19997496 PMC2778997

[77] Carpenter S, Aiello D, Atianand MK, Ricci EP, Gandhi P, Hall LL, Byron M, Monks B, Henry-Bezy M, Lawrence JB, O'Neill LA, Moore MJ, Caffrey DR, Fitzgerald KA. 2013. A long noncoding RNA mediates both activation and repression of immune response genes. Science 341:789–792. doi:10.1126/science.1240925. 23907535 PMC4376668

[78] Nobes CD, Hall A. 1995. Rho, Rac, and Cdc42 GTPases regulate the assembly of multimolecular focal complexes associated with actin stress fibers, lamellipodia, and filopodia. Cell 81:53–62. doi:10.1016/0092-8674(95)90370-4. 7536630

[79] Abmayr SM, Yao T, Parmely T, Workman JL. 2006. Preparation of nuclear and cytoplasmic extracts from mammalian cells. Curr Protoc Mol Biol 12:12.3.1–12.3.13. doi:10.1002/0471141755.ph1203s12. 18265374

[80] Tan BC, Yang CC, Hsieh CL, Chou YH, Zhong CZ, Yung BY, Liu H. 2012. Epigenetic silencing of ribosomal RNA genes by Mybbp1a. J Biomed Sci 19:57–67. doi:10.1186/1423-0127-19-57. 22686419 PMC3407492

[81] Subramaniam D, Ramalingam S, May R, Dieckgraefe BK, Berg DE, Pothoulakis C, Houchen CW, Wang TC, Anant S. 2008. Gastrin-mediated interleukin-8 and cyclooxygenase-2 gene expression: differential transcriptional and posttranscriptional mechanisms. Gastroenterology 134:1070–1082. doi:10.1053/j.gastro.2008.01.040. 18395088

[82] Niranjanakumari S, Lasda E, Brazas R, Garcia-Blanco MA. 2002. Reversible cross-linking combined with immunoprecipitation to study RNA-protein interactions *in vivo*. Methods 26:182–190. doi:10.1016/S1046-2023(02)00021-X. 12054895

[83] Chu C, Qu K, Zhong FL, Artandi SE, Chang HY. 2011. Genomic maps of long noncoding RNA occupancy reveal principles of RNA-chromatin interactions. Mol Cell 44:667–678. doi:10.1016/j.molcel.2011.08.027. 21963238 PMC3249421

