# Supplementary figures and images for: LncRNA XR_001779380 Primes Epithelial Cells for IFN-γ-Mediated Gene Transcription and Facilitates Age-Dependent Intestinal Antimicrobial Defense
Source: mBio. 2021 Sep 7;12(5):e02127-21. doi: 10.1128/mBio.02127-21 (PMC8546593; doi:10.1128/mBio.02127-21)

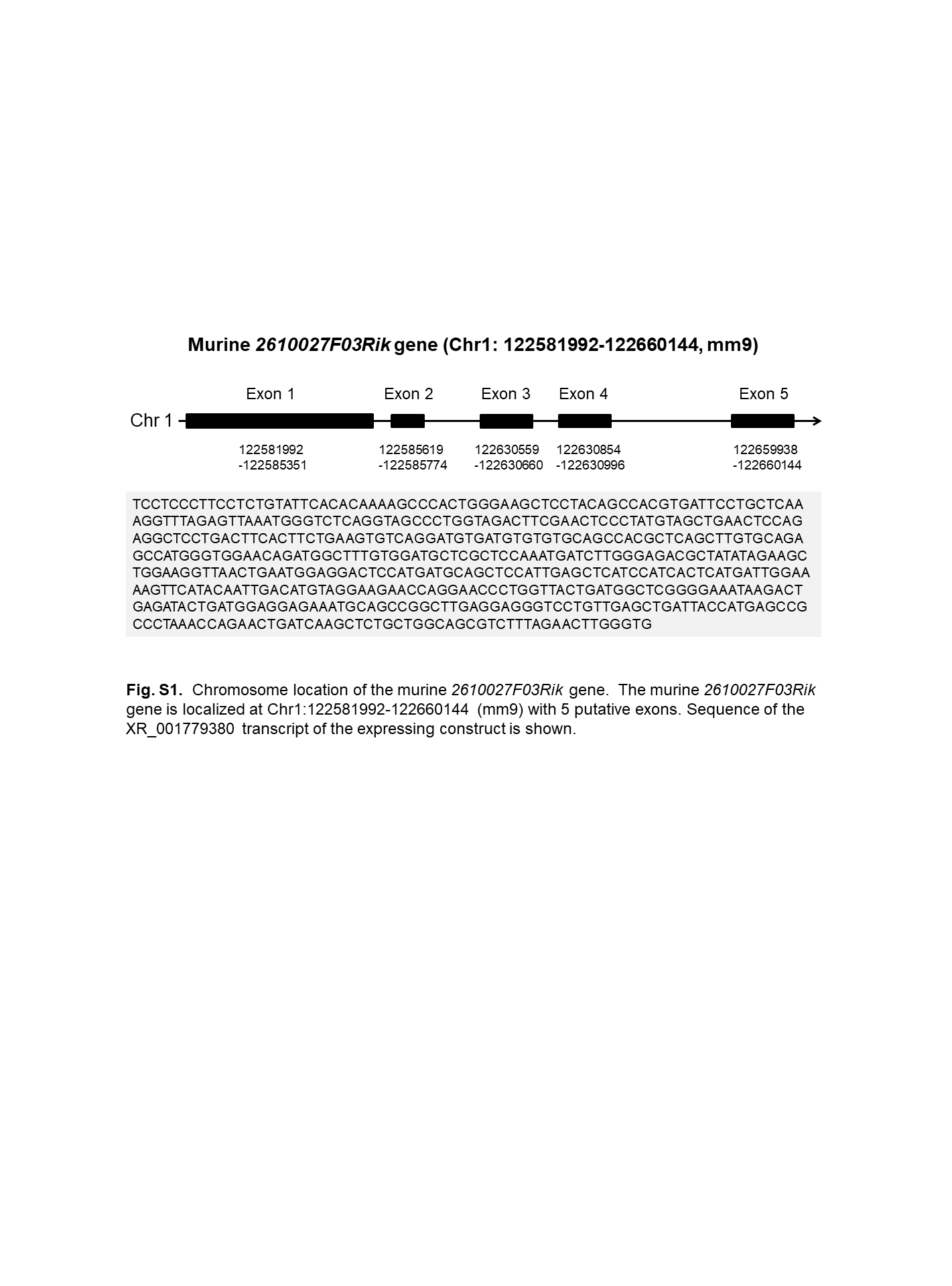

Supplement: FIG S1 [file mbio.02127-21-sf001.tif]

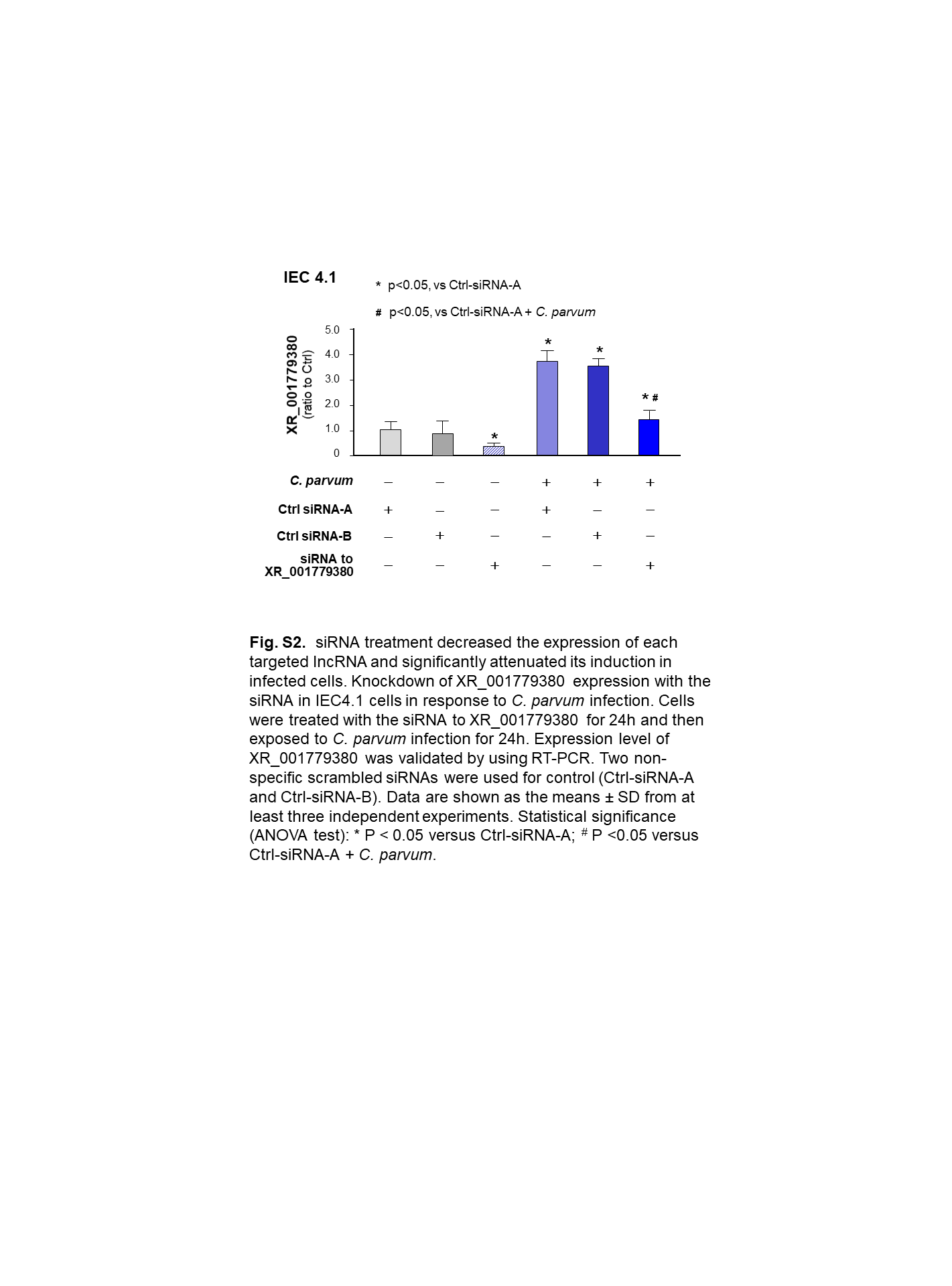

Supplement: FIG S2 [file mbio.02127-21-sf002.tif]

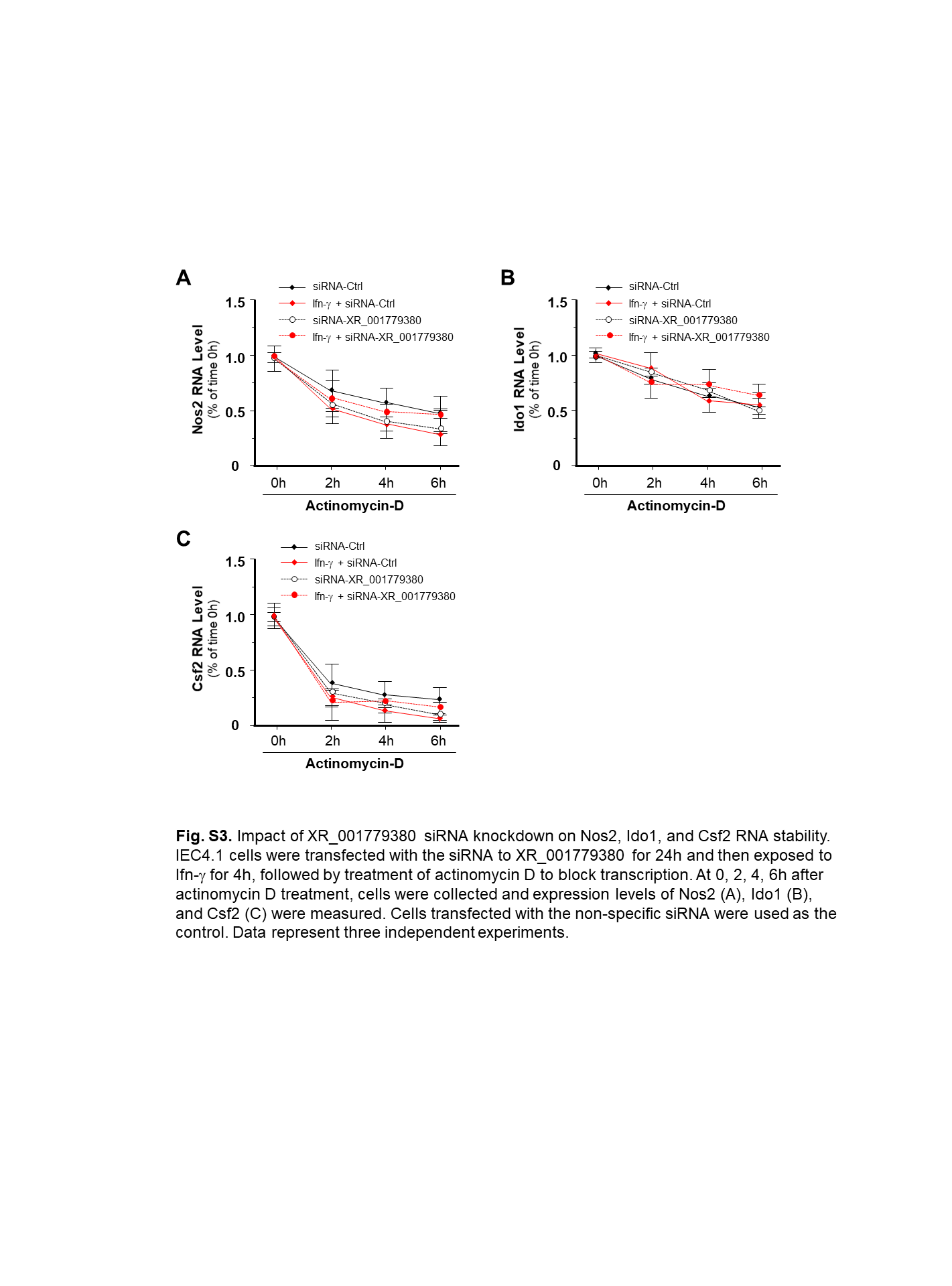

Supplement: FIG S3 [file mbio.02127-21-sf003.tif]

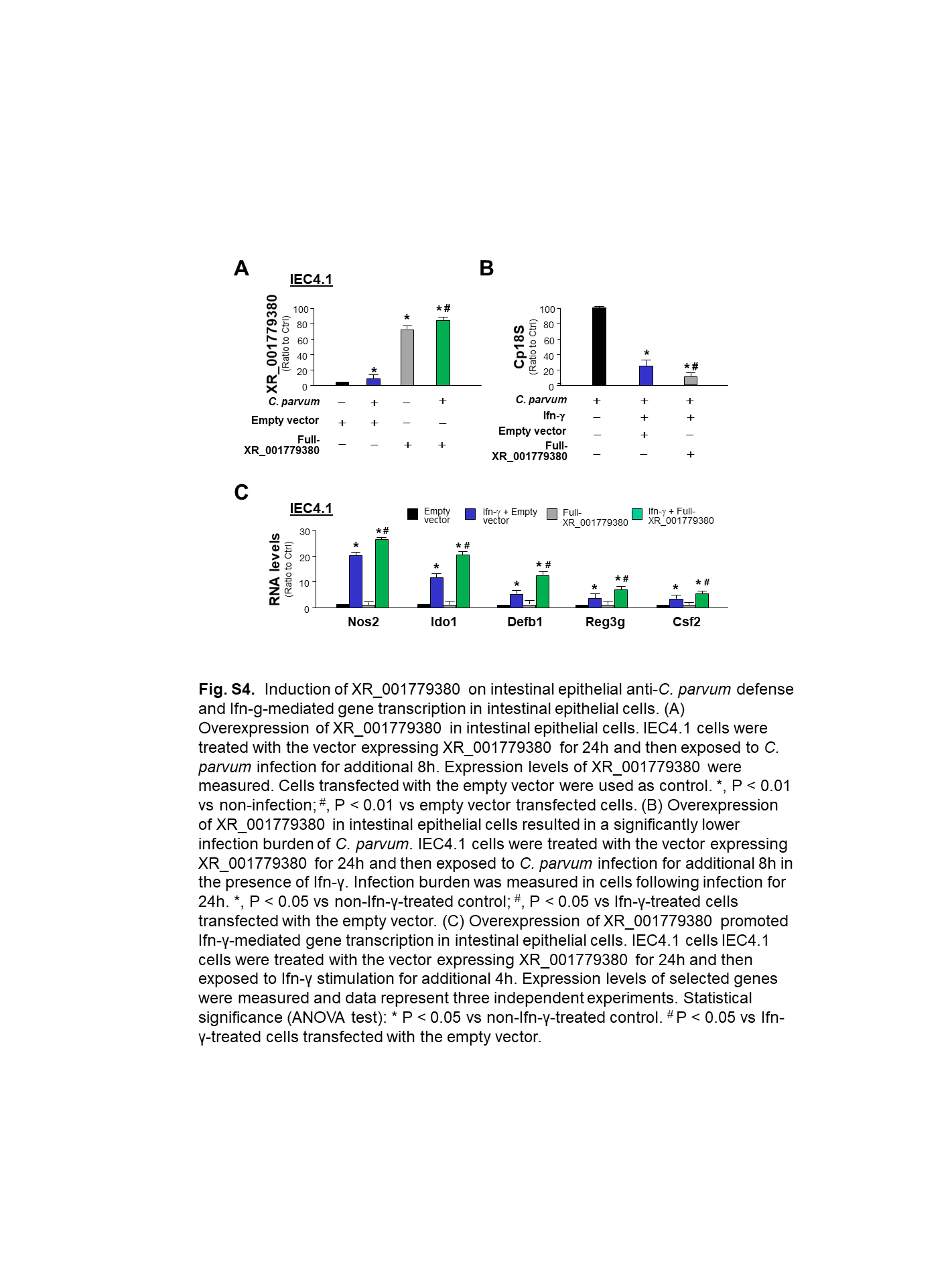

Supplement: FIG S4 [file mbio.02127-21-sf004.tif]

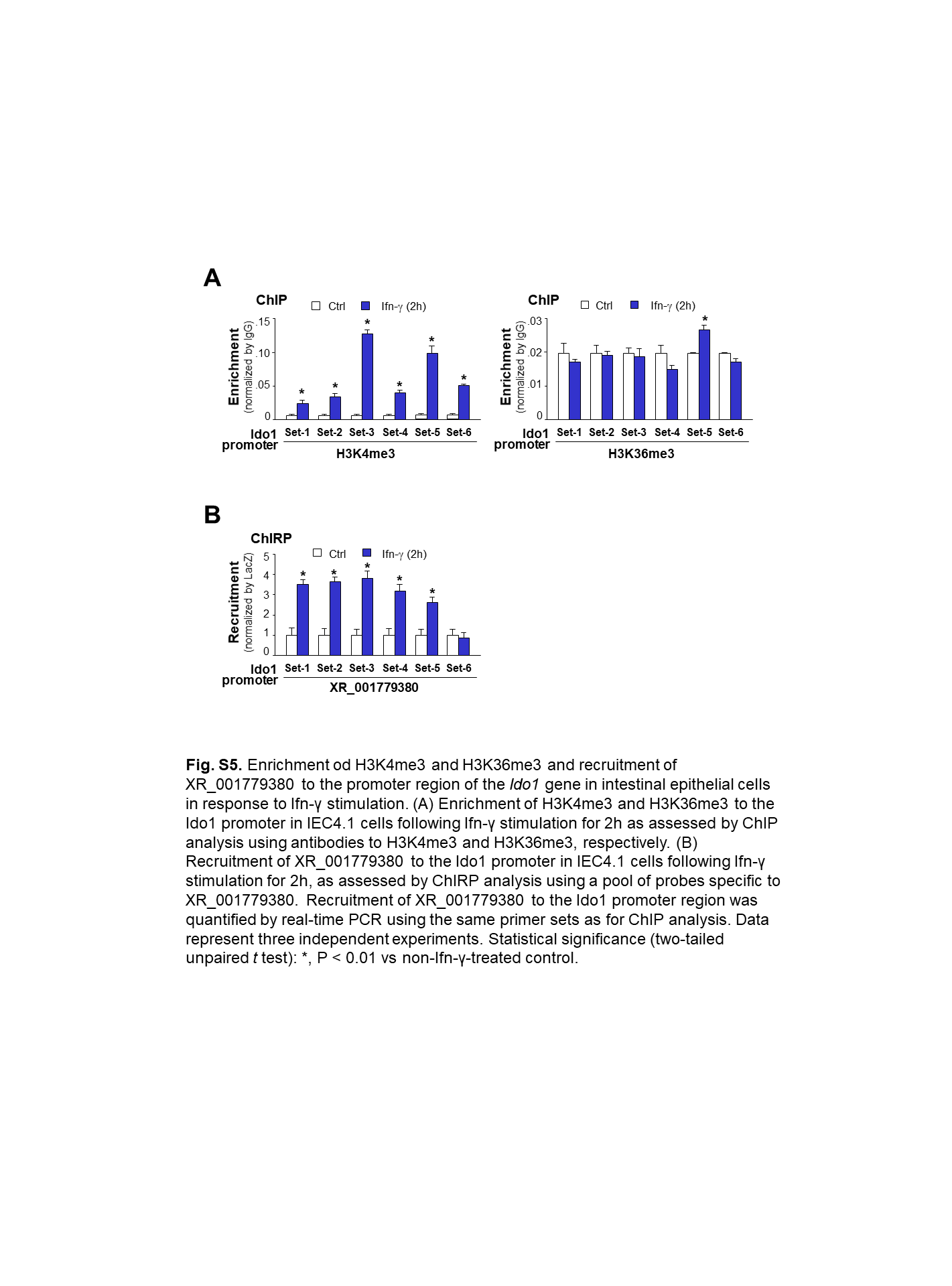

Supplement: FIG S5 [file mbio.02127-21-sf005.tif]

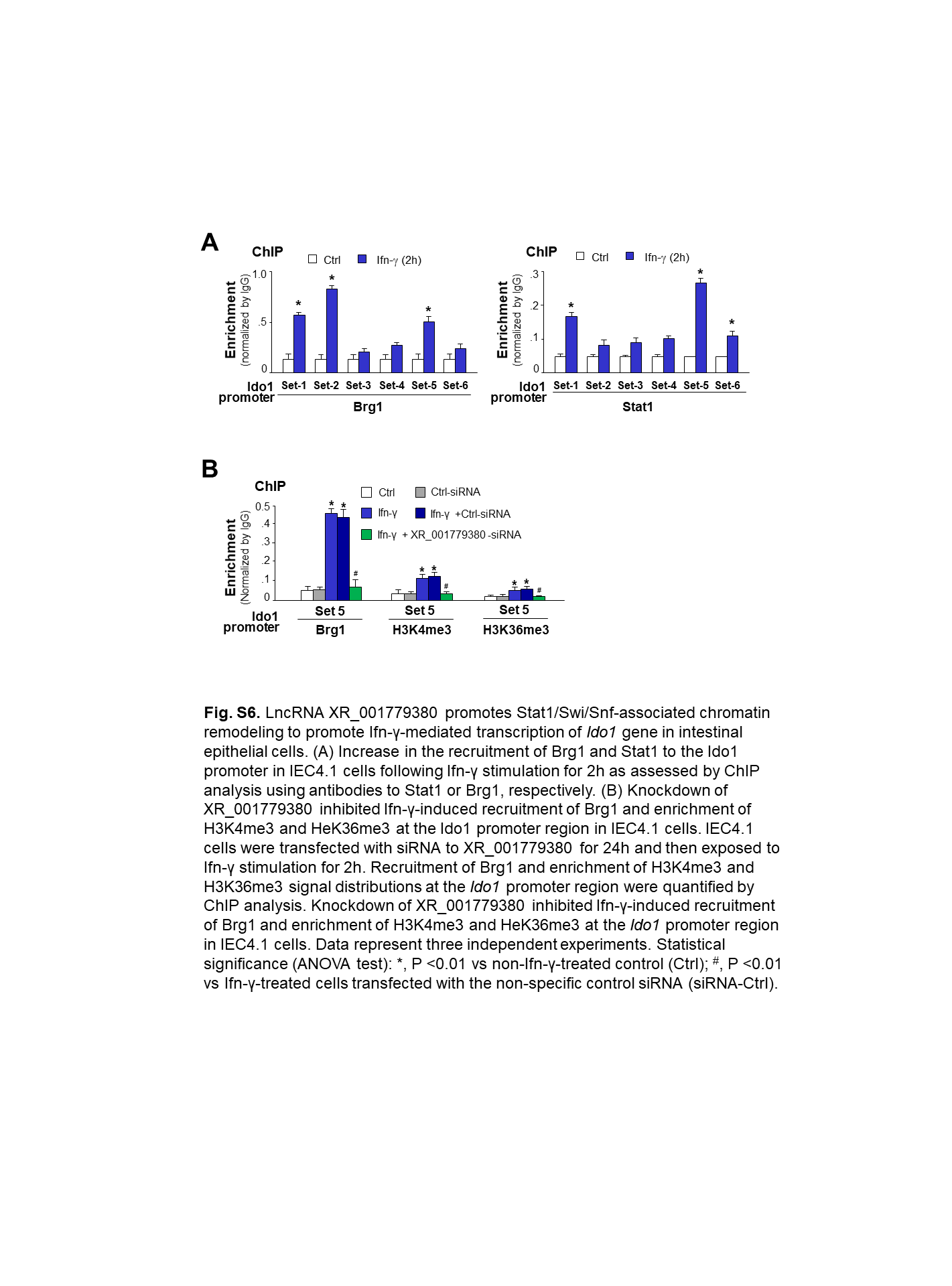

Supplement: FIG S6 [file mbio.02127-21-sf006.tif]

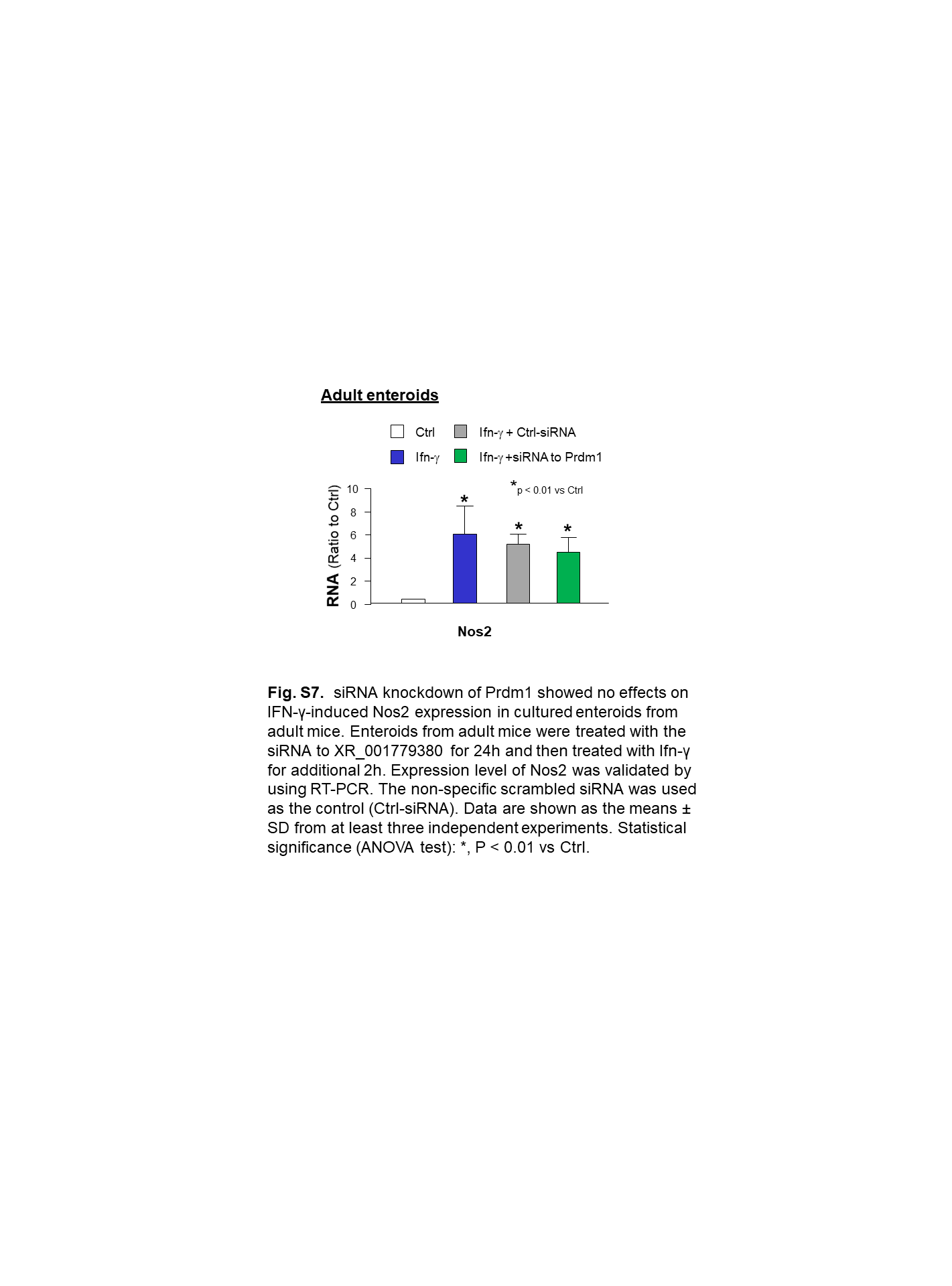

Supplement: FIG S7 [file mbio.02127-21-sf007.tif]
